# Transport of protein disulfide isomerase from the endoplasmic reticulum to the extracellular space without passage through the Golgi complex

**DOI:** 10.1016/j.jbc.2024.107536

**Published:** 2024-07-04

**Authors:** Percillia Victoria Santos Oliveira, Marco Dalla Torre, Victor Debbas, Andrea Orsi, Francisco Rafael Martins Laurindo, Roberto Sitia

**Affiliations:** 1 Division of Genetics and Cell Biology Vita-Salute San Raffaele University and IRCCS San Raffaele Scientific Institute, Milan, Italy; 2 Laboratorio de Biologia Vascular, LIM-64 (Biologia Cardiovascular Translacional), Instituto do Coração (InCor), Hospital das Clinicas HCFMUSP, Faculdade de Medicina, Universidade de São Paulo, São Paulo, SP, Brasil

**Keywords:** protein disulfide isomerase-A1, endoplasmic reticulum, KDEL protein secretion, unfolded protein response, protein trafficking, protein N-glycosylation

## Abstract

Protein disulfide isomerase-A1 (PDIA1) is a master regulator of oxidative protein folding and proteostasis in the endoplasmic reticulum (ER). However, PDIA1 can reach the extracellular space, impacting thrombosis and other pathophysiological phenomena. Whether PDIA1 is externalized *via* passive release or active secretion is not known. To investigate how PDIA1 negotiates its export, we generated a tagged variant that undergoes N-glycosylation in the ER (Glyco-PDIA1). Addition of N-glycans does not alter its enzymatic functions. Upon either deletion of its KDEL ER-localization motif or silencing of KDEL receptors, Glyco-PDIA1 acquires complex glycans in the Golgi and is secreted. In control cells, however, Glyco-PDIA1 is released with endoglycosidase-H sensitive glycans, implying that it does not follow the classical ER-Golgi route nor does it encounter glycanases in the cytosol. Extracellular Glyco-PDIA1 is more abundant than actin, lactate dehydrogenase, or other proteins released by damaged or dead cells, suggesting active transport through a Golgi-independent route. The strategy we describe herein can be extended to dissect how select ER-residents reach the extracellular space.

Dozens of soluble chaperones and enzymes reside in the early secretory pathway to assist the biogenesis of membrane and secretory proteins. An N-terminal signal peptide drives their cotranslational translocation into the endoplasmic reticulum (ER). Most of them possess a KDEL or related tetrapeptide (*e.g.*, RDEL, KTEL, QEDL, and KEEL) at their C termini. This motif prevents their transport beyond the Golgi *via* interactions with cognate KDEL receptors (KRs) (1, 2). A few enzymes that exert their main activity in the ER (*e.g.* Ero1, Prx4, ERAP1, and FGE/SUMF1) lack KDEL motifs. Their localization is guaranteed by indirect binding to KRs, for example, *via* protein disulfide isomerase family proteins such as PDIA1 and/or ERp44 (3, 4). Despite its KDEL ER targeting motif, in fact, PDIA1 has been detected in the supernatants (SNs) and on the surface of platelets (5, 6), activated endothelial cells (6, 7), vascular smooth muscle cells, and other cell types (6, 8). In endothelial cells, for instance, extracellular PDIA1 is estimated as <2% of the total cellular steady-state PDIA1 levels (9). However, the mechanisms of PDIA1 externalization remain incompletely elucidated. This knowledge is important, since extracellular PDIA1 controls many key pathophysiological phenomena, including thrombosis (5, 6, 10), virus entry (11, 12), metalloproteinase activation (13), cell adhesion (6, 14) and vascular remodeling (6, 15). In addition, other ER resident proteins have been described to exert important functional roles extracellularly. For instance, ERp57 (PDIA3), ERp5 (PDIA6), and ERp72 (PDIA4) regulate thrombosis with nonoverlapping roles (5, 10), whilst—particularly upon ER stress—calreticulin (CRT) reaches the cell surface acting as an “eat-me” signal in immunogenic cell death (16). Externalized GRP94 can facilitate tumor cell recognition and immune responses as well (17, 18). Dissecting the mechanisms that regulate the secretion of certain ER-resident proteins, therefore, appears to be particularly relevant.

Two main mechanisms can explain how these proteins elude the surveillance of the three KRs that operate in mammalian cells: active secretion by unknown mechanisms or passive release by damaged or dead cells. The latter can be excluded for ERp44, which is found extracellularly with O-glycans acquired in the Golgi (19). The regulated exposure of the RDEL motif upon zinc deficiency can explain ERp44 secretion (20). So far, for proteins which are not modified posttranslationally like ERp44, evidence for active secretion is based mainly on quantitative reasoning. Thus, if cell damage or death was responsible for the release of PDIA1, the majority of cellular soluble proteins should be equally represented in the extracellular space. Glyceraldehyde-3-phosphate dehydrogenase (GAPDH), lactate dehydrogenase, β-actin, and other abundant proteins are often used to monitor the basal levels of passive release upon loss of membrane integrity. With this strategy, numerous previous studies have documented higher levels of PDIA1 compared to markers of cell damage or death. However, the precise mechanism(s) whereby PDIA1 reaches the extracellular space remain unclear, and several key questions remain: What determines PDIA1 externalization by living cells? Specific secretion mechanisms that elude KRs surveillance or unconventional pathways? Do cell stresses increase its secretion?

We reasoned that a convenient reporter be needed to address the open questions concerning PDIA1 secretion. Considering that differences in the posttranslational modifications between the intracellular and extracellular pools would be proofs for active secretion, we inserted an N-linked glycosylation site in human PDIA1. In this way, by monitoring glycan processing, we were able to follow its trafficking. Surprisingly, our data suggest that small amounts of PDIA1 leave the ER and reach the extracellular space bypassing the Golgi complex.

## Results and discussion

### Building a reporter to follow PDIA1 trafficking from the ER to the extracellular space

**Figure 1:**
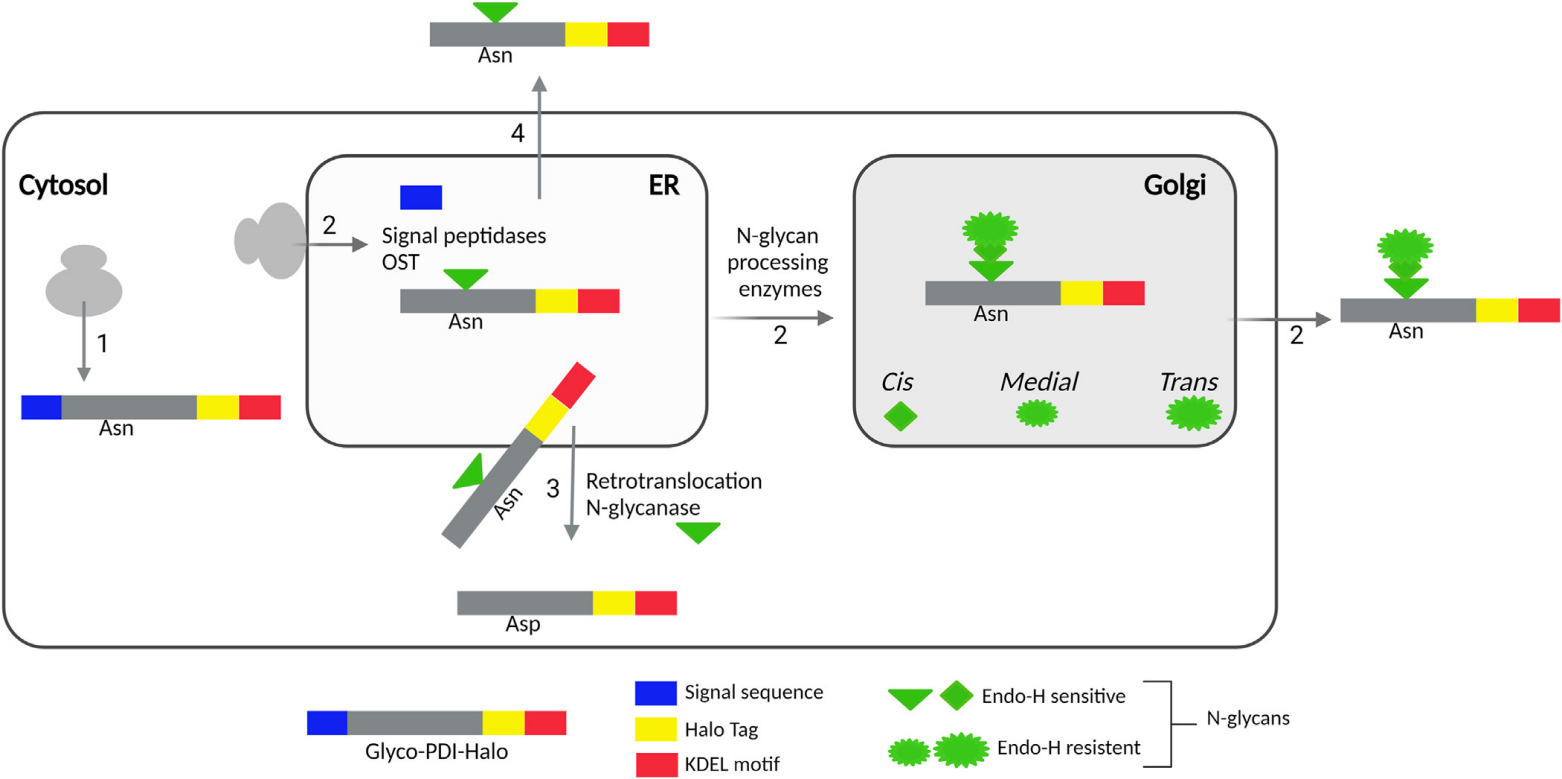
**The strategy**. A N-glycan site was inserted into a human PDIA1 construct equipped with a Halo tag (*yellow box*) to follow the localization of the chimeric protein, based on presence or absence of signal peptide (*blue box*), the degree of sugar processing (differential enzymatic sensitivity, *green symbols*) and the potential asparagine to aspartate conversion by N-glycanases upon retrotranslocation to the cytosol (see details in the text). This strategy can help deciphering the route followed by certain ER resident proteins to the extracellular milieu. Original image created with BioRender. ER, endoplasmic reticulum; Endo-H, endoglycosidase H; PDI, protein disulfide isomerase; PDIA1, protein disulfide isomerase-A1.

To better understand how a subset of PDIA1 molecules reach the extracellular space, we engineered a consensus motif for N-glycosylation into a Halo-tagged human PDIA1, at a similar position normally glycosylated in yeast PDIA1. Our strategy was based on the following notions (Fig. 1): (i) Most secretory glycoproteins have an N-terminal signal peptide that targets them to the ER. If they do not enter the ER, they retain both an unmodified asparagine in the consensus NXS/T sequences and an uncleaved signal peptide (arrow 1) (21, 22); (ii) N-glycans are added to the asparagine upon entry in the ER and then sequentially modified by Golgi-resident enzymes. The type of modifications reveals how far a protein has proceeded along the exocytic pathway (arrows 2) (23); (iii) upon retrotranslocation from the ER to the cytosol, N-glycans are cleaved by resident N-glycanases acting before the proteasome (22). Cleavage of the beta-aspartyl-glycosyl-amine bond converts the target asparagine into an aspartate residue (arrow 3). This strategy would enable us to establish whether the PDIA1 molecules found extracellularly bypassed the ER (retaining the signal peptide) (arrow 1), entered the ER cotranslationally (losing the N-terminal signal peptide), traversed the Golgi, becoming endoglycosidase H (Endo-H) resistant (arrow 2), and/or were dislocated to the cytosol, becoming de-glycosylated and hence more acidic (arrow 3) (24) or leave cells from the ER bypassing the Golgi, (arrow 4); (iv) Halo tags allow powerful imaging and biochemical assays (25, 26).

**Figure 2:**
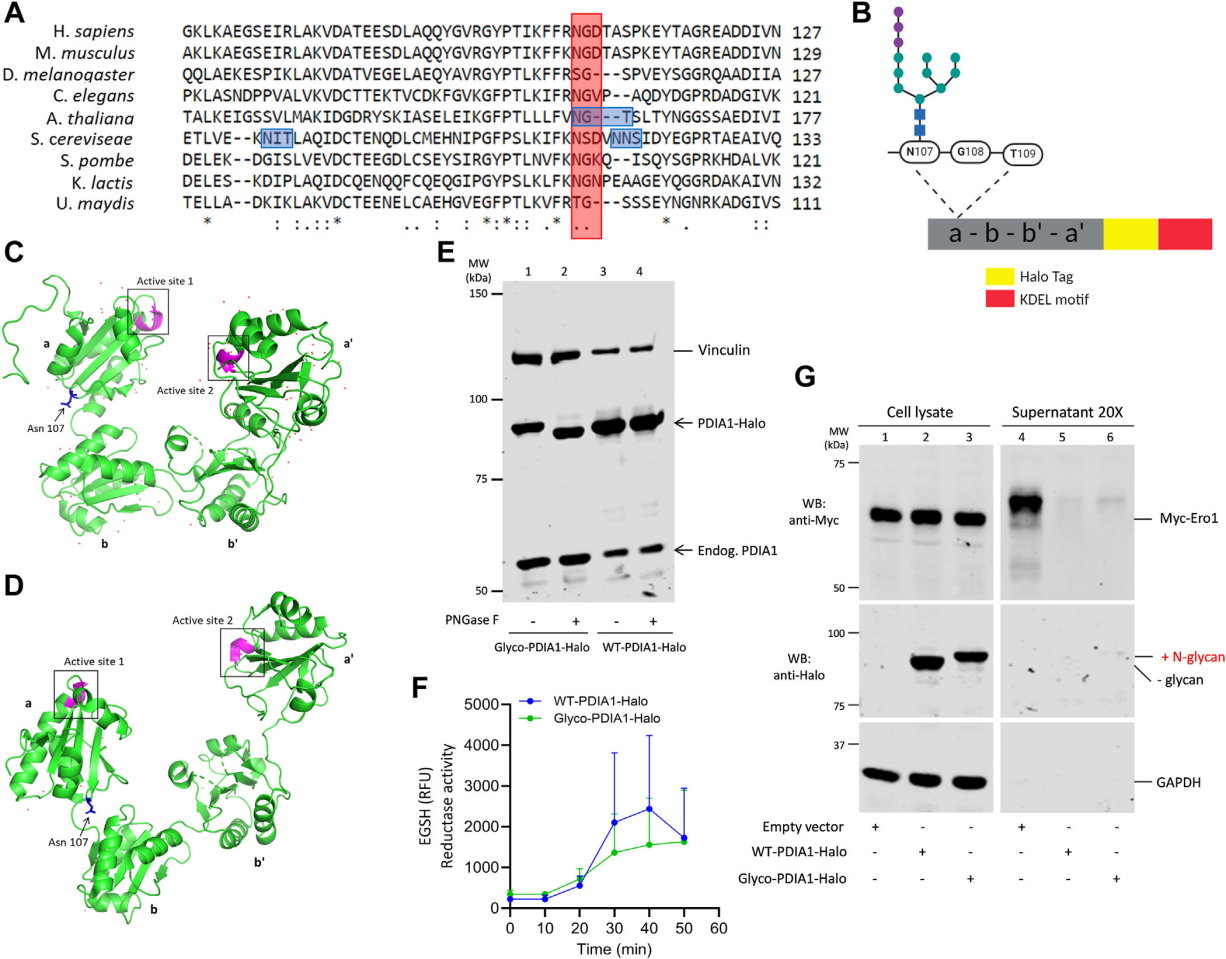
**The construct**. *A*, sequence alignment of PDIA1 from different species shows that the residues chosen to insert a canonical consensus motif for N-glycosylation (N-X-T/S) in human PDIA1 (N107-G108-D109, highlighted in *red*) are not fully conserved. Note that a N-glycan occupies the same position in *Arabidopsis thaliana*, while in *Saccharomyces cerevisiae* one of the overall five glycosylation motifs is found a few residues downstream (highlighted *in light blue*). *B*, schematic representation of PDIA1-Halo used in this study. The *yellow rectangle* at the C terminus represents the Halo-Tag placed between KDEL (*red rectangle*) motif and C-domain. The introduced consensus motif (NGT) is localized in the PDIA1 a-domain. Original image created with BioRender. *C* and *D*, PDIA1 crystal structure reveals that the Asn107 residue is exposed in both reduced (PDB Entry - 4EKZ) (*C*) and oxidized (PDB Entry - 4EL1) (*D*) forms, distant from the active sites highlighted in *magenta*. *E*, lysates from HeLa cells transfected with Glyco-PDIA1-Halo (20 μg), and WT-PDIA1-Halo (5 μg) were digested by PNGase-F, resolved by SDS-PAGE, and membranes immunodecorated with anti-PDIA1 and anti-vinculin antibodies. Note the clear mobility shift in Glyco-PDIA1 (see *black arrow*). *F* and *G*, appending a sugar does not inhibit PDIA1 activity. *F*, reductase activity was evaluated testing the ability of Glyco and WT-PDIA1-Halo purified from cell lysates to reduce the di-eosin-GSSG probe (EGSH). Data represent mean ± SEM from three independent experiments. *G*, expression of either Glyco-PDIA1 or WT-PDIA1 prevent the secretion of overexpressed Ero1α by HeLa transfectants (29). Cells were washed and incubated in Opti-MEM for 4 h. Aliquots of cell lysates (corresponding to 0.1 × 10^6^ cells) and supernatants (corresponding to 2 × 10^6^ cells) were resolved under reducing conditions (6% polyacrylamide gel) and immunoblotted as indicated (n = 2). All lanes come from a single gel. Whole blots are shown in Fig. S9. GSSG, glutathione disulfide; PDIA1, protein disulfide isomerase-A1; RFU, relative fluorescence units.

With this in mind, we replaced aspartate 109 with a threonine residue (Fig. 2) generating an *N107-G108-T109* N-glycan site into a Halo-tagged human PDIA1 (PDI-Halo-D109T) (Fig. S1). The same N-glycan motif is present in *Arabidopsis thaliana* PDIA1 homolog (Fig. 2*A*), suggesting that the site is exposed, and glycan addition does not significantly alter the structure and function of the enzyme. In human PDIA1, these residues are located in the a-domain. Since they are not fully conserved, we did not expect the modification to have disruptive consequences. Importantly, also yeast PDIA1 homolog has N-glycan site near this position, and molecular models predict that Asn-107 residue be exposed to the solvent and distant from the active sites in both the reduced or oxidized PDIA1 isoforms (Fig. 2, *C* and *D*). Thus, the covalent linkage of the oligosaccharide GlcNac_2_Man_9_Glu_3_ to this residue should not impact PDIA1 conformation and activity (see below). Since N-glycans are necessary but not sufficient for N-glycosylation to occur, we first ensured that the motif inserted was recognized by oligosaccharyl transferase. When expressed in HeLa cells, PDIA1-Halo-D109T migrated more slowly than its T109 counterpart. Consistent with the presence of an N-glycan, treatment with PNGase-F (an enzyme capable of removing also complex N-glycan moieties from glycoproteins, see below) increased its mobility on SDS-PAGE (Fig. 2*E*, compare lanes 1–2). As expected, WT-PDIA1-Halo, that does not carry N-glycosylation sites, did not show any changes in electrophoretic mobility (Fig. 2*E*, lanes 3–4).

Next, to verify whether our reporter constructs maintained their functionality, we evaluated their ability to reduce di-eosin-glutathione disulfide (GSSG) *in vitro* (27, 28) and to bind Ero1α and prevent its secretion by living HeLa cells (29, 30). Clearly, the presence of an N-glycan affected neither reductase activity (Fig. 2*F*) nor retention of overexpressed Ero1α (Fig. 2*G*, compare lanes 4–6). Interesting, co-expression of Ero-1α inhibited PDIA1 secretion suggesting a client-induced retention as shown previously for ERp44 (19). Thus, PDIA1-Halo-D109T presents characteristics of an accurate tool to investigate PDIA1 trafficking. For brevity, we will hereafter refer to it as Glyco-PDIA1.

### Extent of Glyco-PDIA1 sugar processing

**Figure 3:**
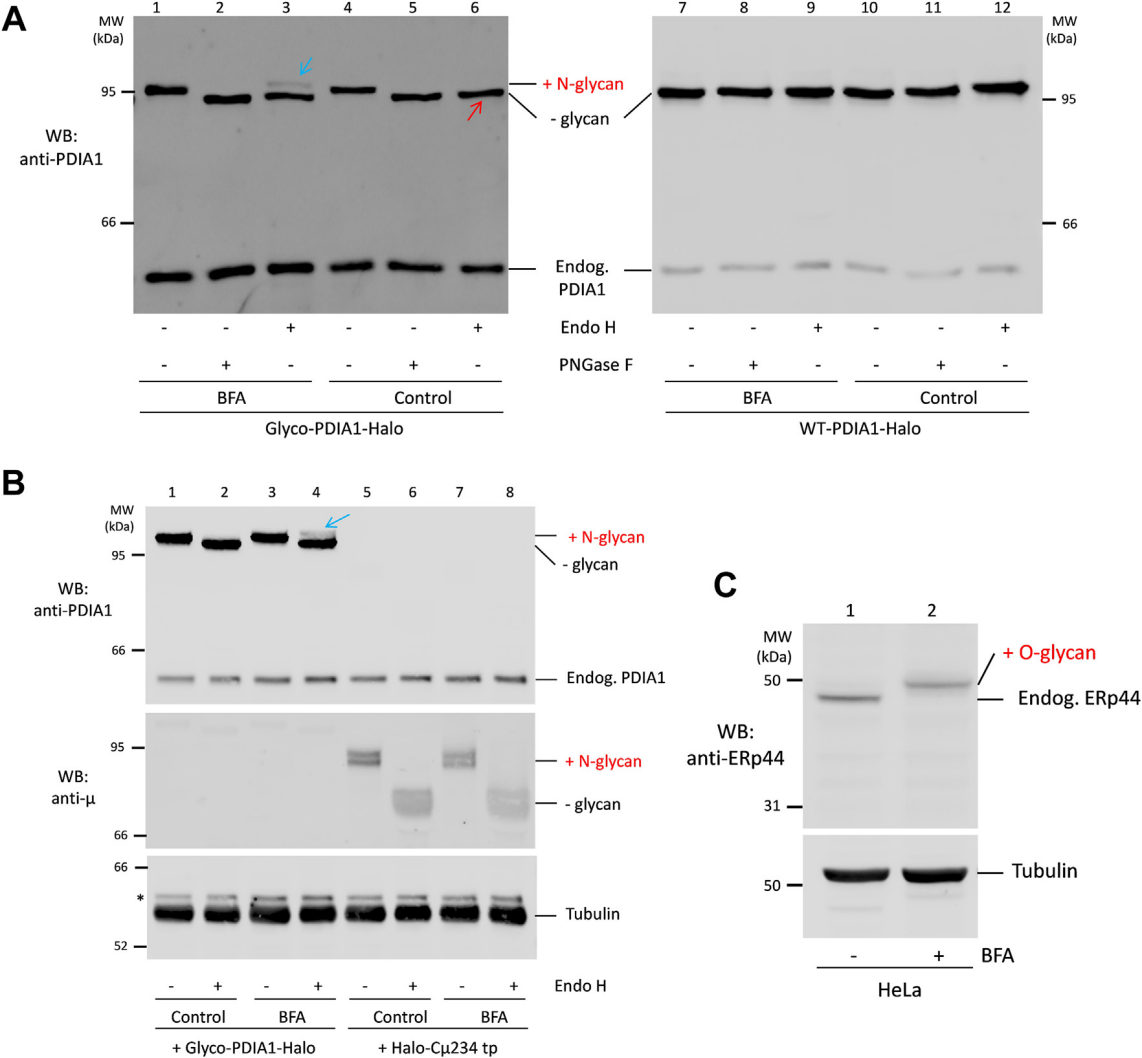
**Extent of Glyco-PDIA1 sugar processing**. *A*, the indicated HeLa transfectants were cultured with or without BFA (25 μg/ml) in Opti-MEM. After 4.5 h, aliquots of cell lysates (10 μg total protein) were treated with Endo-H or PNGase-F, resolved under reducing conditions (8% polyacrylamide gel) and immunoblotted with anti-PDIA1 antibodies. The *red* and *blue arrows* point to enzyme-sensitive or resistant PDIA1 molecules, respectively (n = 3). As expected, the mobility of WT-PDIA1 does not change after enzymatic digestion. All lanes come from a single gel. Whole blots are shown in Fig. S10. *B*, HeLa cells transiently transfected with Halo-Cμ234 tp (a shortened variant of Ig-μ chains) and Glyco-PDIA1 and treated and processed as in panel (*A*). Blots were decorated with anti-μ, anti-PDIA1, and anti-tubulin (n = 3). The *blue arrow* points to Endo-H resistant PDIA1 molecules. (∗) indicates endogenous PDIA1 band from the previous staining. *C*, HeLa cells were processed as in panel (*A*) and resolved under reducing conditions (4–12% precast polyacrylamide gel). Blots were decorated with anti-ERp44 and anti-tubulin antibodies (n = 3). The slower gel mobility in lane 2 with respect to lane 1 reflects ERp44 O-glycosylation after BFA exposure (19). BFA, Brefeldin A; Endo-H, endoglycosidase H; PDIA1, protein disulfide isomerase-A1.

To investigate the diversity of N-glycan structures present in Glyco-PDIA1, the lysates of HeLa cells expressing Glyco-PDIA1 were treated with Endo-H or N-glycanase (PNGase- F). The former cuts only immature, high mannose or hybrid-type N-glycans, while the latter cuts all N-linked sugar moieties. Treatment with either Endo-H or PNGase-F decreased the molecular weight of Glyco-PDIA1 but not of WT-PDIA1 (Fig. 3*A*, compare lanes 4–6 and 10–12). To generate complex-type glycan structures resistant to Endo-H, glycoproteins must be processed by enzymes residing in the medial and trans-Golgi stacks. Thus, sensitivity to Endo-H indicates that most Glyco-PDIA1 did not proceed beyond the *cis*-Golgi, as in fact expected for a KDEL-bearing protein.

In many experiments, Glyco-PDIA1 was expressed less than its WT counterpart (*e.g.*, Fig. 3*A*), raising the possibility that the presence of a sugar targeted it to degradation. However, radioactive pulse-chase assays revealed that Glyco-PDIA1 was as stable as its nonglycosylated counterpart. Also, inhibitors of proteasomal activity (bortezomib), of glycoprotein retrotranslocation to the cytosol (kifunensine, Kif), and of the calnexin/CRT cycle (castanospermine) did not affect their stability (Fig. S2). WT and Glyco-PDIA1 displayed an intense reticular distribution, colocalizing with CRT (Fig. S3*A*). Their weak staining in GM130-positive *cis*-Golgi stacks (Fig. S3*B*) indicated that both our reporters accumulate mainly in the ER.

Brefeldin A (BFA) induces the retrograde transport of Golgi enzymes to the ER. As previously observed (19), BFA treatment promoted ERp44 O-glycosylation (Fig. 3*C*). Likewise, a fraction of Glyco-PDIA1 exhibited lower electrophoretic mobility and Endo-H resistance after BFA exposure (Fig. 3*A*, lanes 2–3 and Fig. 3*B*, compare lanes 2–4). Thus, our probe can be processed to complex glycans. Unexpectedly, however, this was not the case for a shortened variant of immunoglobulin (Ig)-μ chains (Halo-Cμ234tp (31)), which in our experiments were not significantly processed in the presence of BFA (Fig. 3*B*, compare lanes 6 and 8).

### Glyco-PDIA1 as a tool to monitor passage through Golgi

**Figure 4:**
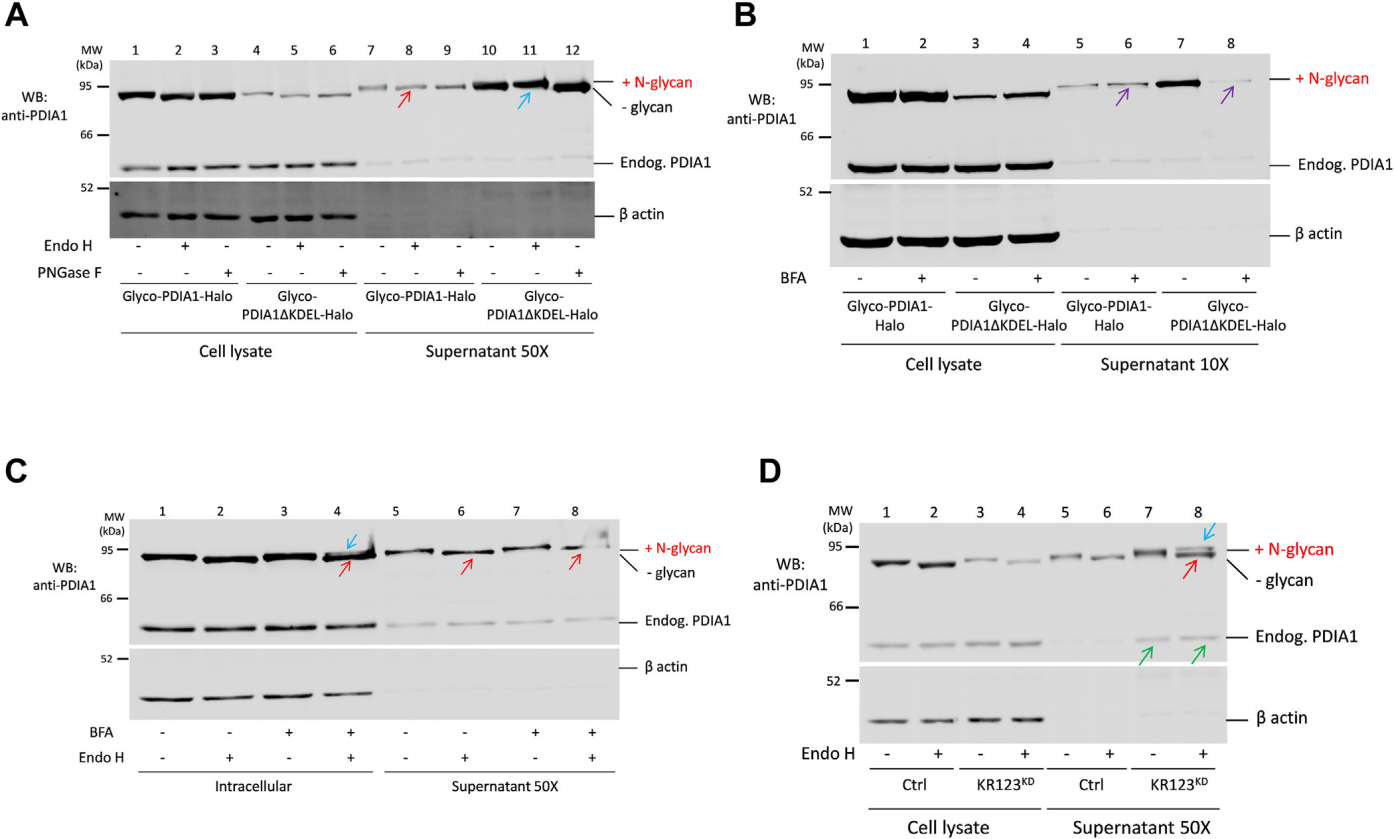
**Glyco-PDIA1 as a tool to monitor Golgi passage**. *A*, the indicated HeLa transfectants were washed and incubated in Opti-MEM for 4 h. Aliquots of cell lysates (corresponding to ∼2 × 10^4^ cells, approximately 10 μg total protein) and supernatants (corresponding to 1 × 10^6^ cells) were treated with Endo-H and PNGase-F, resolved by reducing SDS-PAGE (8% polyacrylamide gel), and immunoblotted with the indicated antibodies (n = 3). The *red* and *blue arrows* points to Endo-H sensitive or resistant PDIA1 molecules, respectively. As expected PNGase-F cuts also Endo-H resistant oligosaccharides. *B*, HeLa cells transiently expressing Glyco-PDIA1 or Glyco-PDIA1ΔKDEL were washed and incubated in Opti-MEM for 4.5 h in the presence or absence of BFA (25 μg/ml). Aliquots of cell lysates (corresponding to 0.1 × 10^6^ cells) and supernatants (corresponding to 1 × 10^6^ cells) were collected and analyzed as above (n = 3). *Purple arrows* point to BFA treated samples. *C*, HeLa cells transiently expressing Glyco-PDIA1 were treated as described in panel (*B*) and digested with Endo-H. Aliquots of cell lysates (corresponding to ∼2 × 10^4^ cells, 10 μg) and supernatants (corresponding to 1 × 10^6^ cells) were resolved under reducing conditions and immunoblotted with the indicated antibodies (n = 3). *D*, HeLa cells were silenced with KDEL receptors 1-, 2-, and 3-specific triplex or control (Ctrl) reagents as indicated and forced to transiently express Glyco-PDIA1. Forty-eight hours after transfection (72 h after silencing), cells were incubated in Opti-MEM for 4 h. Aliquots of cell lysates (corresponding to ∼2 × 10^4^ cells) and supernatants (1 × 10^6^ cells) were treated with or without Endo-H, resolved under reducing conditions (8% polyacrylamide gel), and blots immunodecorated with anti-β actin or anti-PDIA1 (n = 3). The *red* and *blue* arrows point to Endo-H-sensitive or -resistant PDIA1 molecules. *Green arrows* point to secreted endogenous PDIA1 (n = 3). BFA, Brefeldin A; Endo-H, endoglycosidase H; PDIA1, protein disulfide isomerase-A1.

Since Glyco-PDIA1 can become Endo-H resistant, it could be used to investigate how a fraction of PDIA1 molecules—the bulk of which reside in the ER—negotiate their release from living cells. Several mechanisms concur in determining the accumulation of PDIA1 in the ER. First, its N-terminal signal sequence targets it there. Second, PDIA1 interacts with other resident proteins of the ER matrix (32, 33) but not with cargo receptors like ERGIC53, explaining why it is secreted more slowly than ERp44 upon deletion of their C-terminal motifs (26, 30). Third, its C-terminal KDEL motif mediates retrieval of PDIA1 molecules that reach the Golgi *via* KRs (4). Accordingly, large amounts of the reporter were secreted upon deletion of the KDEL motif (ΔKDEL). Importantly, secreted Glyco-PDIA1ΔKDEL was largely Endo-H resistant (Fig. 4*A*, compare lanes 10 and 11, see blue arrow), as expected for a complete passage through the Golgi complex. In contrast, most intracellular molecules remained Endo-H sensitive (Fig. 4*A*, lane 5), suggesting rapid transport to the extracellular space beyond the *trans*-Golgi. These findings confirmed that Glyco-PDIA1ΔKDEL becomes Endo-H resistant upon passage through the Golgi on its way out from the cell.

As expected, much smaller but significant amounts of Glyco-PDIA1 were instead detected in the SNs of KR competent cells. Importantly, these molecules were Endo-H sensitive (Fig. 4*A* lane 8, see red arrow). Since Endo-H resistance can be acquired upon KDEL deletion, these findings suggested that when the KDEL-dependent retrieval system is operating, a fraction of PDIA1 molecules can still be secreted but bypassing the Golgi (Fig. 4*A*, compare lanes 7 and 8). As predicted, both Glyco-PDIA1 and the corresponding ΔKDEL mutant were sensitive to PNGase-F (Fig. 4*A*, lanes 9 and 12).

To further confirm the Golgi bypass route of Glyco-PDIA1, cells were treated with BFA, a drug known to block ER to Golgi transport. Strikingly, BFA treatment blunted almost completely Glyco-PDIA1ΔKDEL secretion (Fig. 4*B*, compare lanes 7 and 8), but it did not significantly alter the amount of secreted Glyco-PDIA1 (Fig. 4*B*, compare lanes 5 and 6). Notably, secreted Glyco-PDIA1 after BFA exposure remained largely Endo-H sensitive (Fig. 4*C*, compare lanes 6 and 8). Thus, the pool of secreted Glyco-PDIA1 was not modified by the Golgi enzymes that were relocated into the ER, even if Glyco-PDIA1 was partially Endo-H resistant in the lysates of BFA-treated cells (Fig. 4*C*, lane 4, see blue arrow). These data may suggest that not the entire ER fuses with the Golgi upon BFA treatment.

Next, we used visual pulse chase assays (Fig. S4*A*) to compare the basal secretory rate of WT and Glyco-PDIA1 with another PDI family member, ERp44 (26). In all three cases, the intracellular TMR (Halo ligand) signal decreased over time (Fig. S4, *B* and *D*), accumulating extracellularly (Fig. S4, *B* and *C*). There was no significant difference between WT and Glyco-PDIA1 secretion rates over time. As previously noted, however, PDIA1ΔKDEL was secreted more slowly than ERp44ΔRDEL, whose anterograde transport is facilitated by ERGIC-53 (26, 30).

The above results suggested that most of the PDIA1 molecules that reach the Golgi are retrieved to the ER by KRs, but some of them find an alternative way to go out. Accordingly, silencing the three KRs (KR123^KD^) induced abundant secretion of Glyco-PDIA1. Upon KR123^KD^, a fraction of secreted Glyco-PDIA1 remained Endo-H sensitive (Fig. 4*D*, lane 8, see red arrow), while some molecules underwent further sugar processing in the Golgi (Fig. 4*D*, lane 8, see blue arrow). A general defect in N-glycosylation due to KR silencing was excluded as glycan processing of secreted Ig-μ chains was not affected (Fig. S5). As expected, endogenous PDIA1 was detectable in the SNs upon KR silencing (Fig. 4*D*, lanes 7 and 8, see green arrows). Downregulation of all three KRs was monitored by RT-PCR (Fig. S6).

The above observations raise interesting speculations regarding PDIA1 trafficking. In the absence of a KR-binding motif, Glyco-PDIA1ΔKDEL or Glyco-PDI mutants (see below) are entirely modified by Golgi enzymes, becoming fully Endo-H resistant. Thus, the incomplete Endo-H resistance of Glyco-PDIA1 upon KR123^KD^ implies that part of it might reach the extracellular space avoiding the Golgi, as does basal PDIA1 secretion in KR competent cells. Why does not all secreted Glyco-PDIA1 acquire complex glycans upon KR^KD^? Evidence is accumulating that KDEL-bearing proteins are not uniformly distributed along the early secretory pathway, depending on the balance between static retention, forward movement and retrieval (4). In such a scenario, at least two pools of PDIA1 likely reside in the early secretory pathway: an abundant ‘static’ one, as part of the matrix that assists nascent cargo proteins in the rough ER, and a smaller pool of ‘mobile’ molecules that reach the *cis*-Golgi and are retrieved by KRs. This pool should yield most Glyco-PDIA1 molecules that become Endo-H resistant as well as endogenous PDIA1 found in SNs upon KR^KD^.

**Figure 5:**
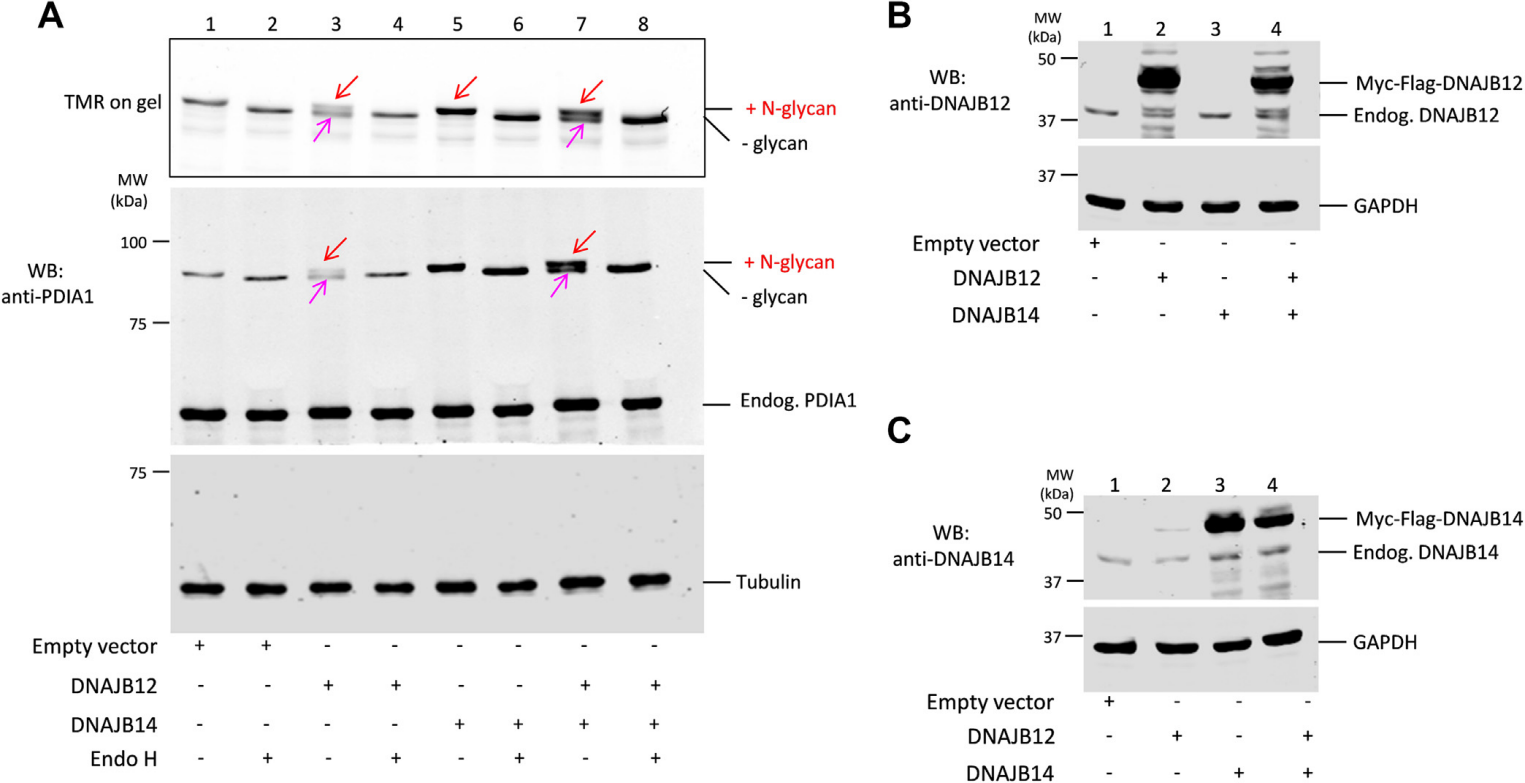
**Evidence that DNAJB12 overexpression induces Glyco-PDIA1 transport from the ER to cytosol.** In panels (*A*–*C*)cells were prepared in the same way. HeLa cells transiently co-expressing Glyco-PDIA1 together with Flag-Myc-tagged DNAJB12 and DNAJB14 or the empty vector were washed and incubated with a fluorescent ligand (tetramethylrhodamine, TMR) to label Glyco-PDIA1-Halo molecules for 4 h in Opti-MEM. *A*, aliquots of cell lysates (20 μg) were treated with Endo-H, resolved by reducing SDS-PAGE (6% polyacrylamide gel) and the Halo/TMR fluorescent signals detected in gels (532 nm). After that, proteins were transferred to membranes and decorated with anti-tubulin and anti-PDIA1 (n = 3). The *red* and *pink arrows* point to glycosylated PDIA1 (Endo-H sensitive) or deglycosylated PDIA1 molecules. *B* and *C*, aliquots of cell lysates (25 μg) were resolved by reducing SDS-PAGE (12% polyacrylamide gel). Blots were immunodecorated with (*B*) anti-DNAJB12 and anti-GAPDH or (*C*) anti-DNAJB14 and anti-GAPDH (n = 3). ER, endoplasmic reticulum; GAPDH, glyceraldehyde-3-phosphate dehydrogenase; Endo-H, endoglycosidase H; PDIA1, protein disulfide isomerase-A1.

Particularly under conditions of ER stress, some resident chaperones including PDIA1 can be retrotranslocated to the cytosol. This phenomenon, dubbed ER to cytosol signaling (ERCYS) or ER reflux (34, 35), can be facilitated by two ER membrane proteins, DNAJB12 and DNAJB14, together with their cytosolic interactor SGTA (36). Thus, we overexpressed Myc-Flag–tagged DNAJB12 and DNAJB14 in order to assess their impact on Glyco-PDIA1 relocalization. When co-expressed in cells with Glyco-PDIA1, DNAJB12 but not DNAJB14 induced deglycosylation of the reporter (Fig. 5*A*, compare lanes 3, 5, and 7). Accordingly, a deglycosylated PDIA1 band (pink arrows) with higher mobility appears below glycosylated PDIA1 band (red arrows) when DNAJB12 was expressed (Fig. 5*A*, compare lanes 1, 3, and 7) indicating that a fraction of Glyco-PDIA1 was retrotranslocated from the ER to the cytosol where N-glycans were removed by resident N-glycanases. As expected, Endo-H treatment resulted in a single band corresponding to complete removal of Glyco-PDIA1 immature N-glycans in all conditions (Fig. 5*A*, lanes 2–4, 6–8). As confirmed by Western blotting analyses, DNAJB12 and DNAJB14 were expressed at similar levels (Fig. 5, *B* and *C*). Taken together, these findings indicate that DNAJB12 is more active than DNAJB14 to induce ER-to-cytosol Glyco-PDIA1 transport. It agrees with previous data showing that DNAJB12 downregulation tend to diminish PDIA1 release into cytosolic-enriched fractions during ER stress, while its overexpression augments it even in the absence of ER stress (36).

Thus, the presence of sugars on extracellular Glyco-PDIA1 argues against retrotranslocation from the ER to cytosol before externalization. At present, the available data do not allow to discriminate between selective transport or passive release of Glyco-PDIA1 in KR competent cells. In cultured endothelial cells, PDIA1 release was largely unaffected by BFA, suggesting that even in circumstances involving negligible cell death, PDIA1 can leave the cell *via* Golgi-independent route(s) (8).

### Glyco-PDIA1 as a tool to monitor Golgi passage: KDEL variants

**Figure 6:**
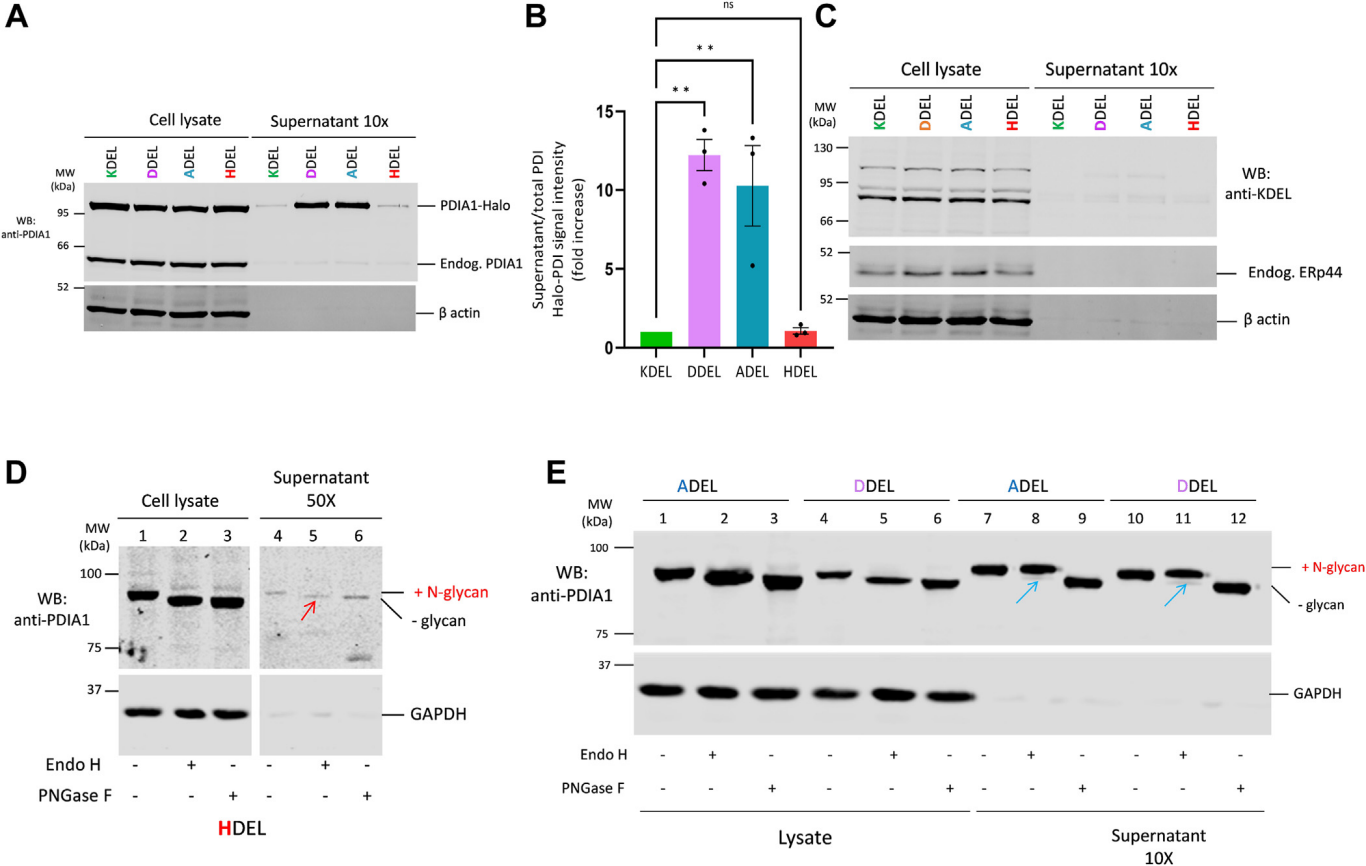
**Weak interaction with KR promotes Glyco-PDIA1 Golgi transport**. *A* and *B*, HeLa cells transiently expressing Glyco-PDIA1 KDEL variants (ADEL, DDEL, and HDEL) were washed and incubated in Opti-MEM for 4 h. *A*, aliquots of cell lysates (corresponding to 0.1 × 10^6^ cells) and supernatants (corresponding to 1 × 10^6^ cells) were collected. After supernatants concentration through TCA precipitation, both were resolved and immunoblotted with anti-PDIA1 or anti-β actin. *B*, the graph shows a quantification of secreted PDIA1 relative to its intracellular pool. Bars represent means ± SEM. Statistical analysis was performed using ANOVA one-way, Dunnett’s post-test. ∗∗*p* < 0.002. ns: nonsignificant (n = 3). *C*, overexpression of HDEL, DDEL, or ADEL PDIA1 does not saturate KR-mediated retrieval. Samples were prepared as described in panel (*A*) and blots decorated with anti-KDEL, anti-ERp44, or anti-β actin. *D* and *E*, HeLa cells transiently expressing Glyco-PDIA1-HDEL (*D*), Glyco-PDIA1-ADEL, or DDEL (*E*) were prepared as described above. Aliquots of cell lysates (∼2 × 10^4^ cells) and supernatants (corresponding to 1 × 10^6^ cells) were treated with Endo H and PNGase F, resolved, and decorated with anti-GAPDH and anti-PDIA1 (n = 3). The *red* and *blue arrows* point to Endo-H-sensitive or -resistant PDI molecules, as above. In panel (*D*), all lanes come from a single gel. Whole blots are shown in Fig. S11. Endo-H, endoglycosidase H; GAPDH, glyceraldehyde-3-phosphate dehydrogenase; KR, KDEL receptor; PDIA1, protein disulfide isomerase-A1.

It was previously shown that canonical ER retrieval signals bind to KRs with different affinities. HDEL has the highest affinity to KR followed by KDEL and RDEL variants, while DDEL and ADEL are ineffective as retrieval signals in human cells (37). Thus, to further explore our tool, we generated Glyco-PDIA1 bearing HDEL, ADEL, and DDEL retrieval signals to investigate whether their interaction with KRs could affect their ability to exit the ER. When expressed in cells, HDEL was secreted as much as the canonical Glyco-PDIA1-Halo-KDEL (Endo-H sensitive, Fig. 6*D*), whereas ADEL and DDEL variants were efficiently secreted (Fig. 6, *A* and *B*). Notably, these variants were secreted in an Endo-H–resistant form (Fig. 6*E*, lanes 8 and 11, see blue arrows) indicating that they did flow through the Golgi. Glyco-PDIA1-HDEL was not fully retained. Even if largely overexpressed, it did not induce significant leakage of endogenous PDIA1 or other KDEL-bearing proteins (Fig. 6*C*). These findings support the notion that a small fraction of PDIA1 molecules leave the static ER pool to engage KR binding and confirm that efficient retrieval limits the arrival of Endo-H resistant forms into the extracellular milieu. They also suggest that KR engagement may promote the Golgi bypass route. Clearly, variants unable to bind KR enter the Golgi and are secreted with complex sugars. It is presently unclear why more Endo-H-sensitive PDIA1 molecules are secreted when KRs are silenced than when the KDEL motif is deleted or replaced. When retrieval is ineffective, many soluble chaperones and enzymes leave the cell, possibly weakening the folding capacity and inducing ER stress. Moreover, KR silencing is rarely complete, and some mobile PDIA1 molecules may be still retrieved and able to bypass the Golgi in their way out. Altogether, these findings argue against loss or hindrance of the KDEL motif as a reason for PDIA1 release.

### Adaptive and maladaptive ER stress display opposite effects on PDIA1 secretion

**Figure 7:**
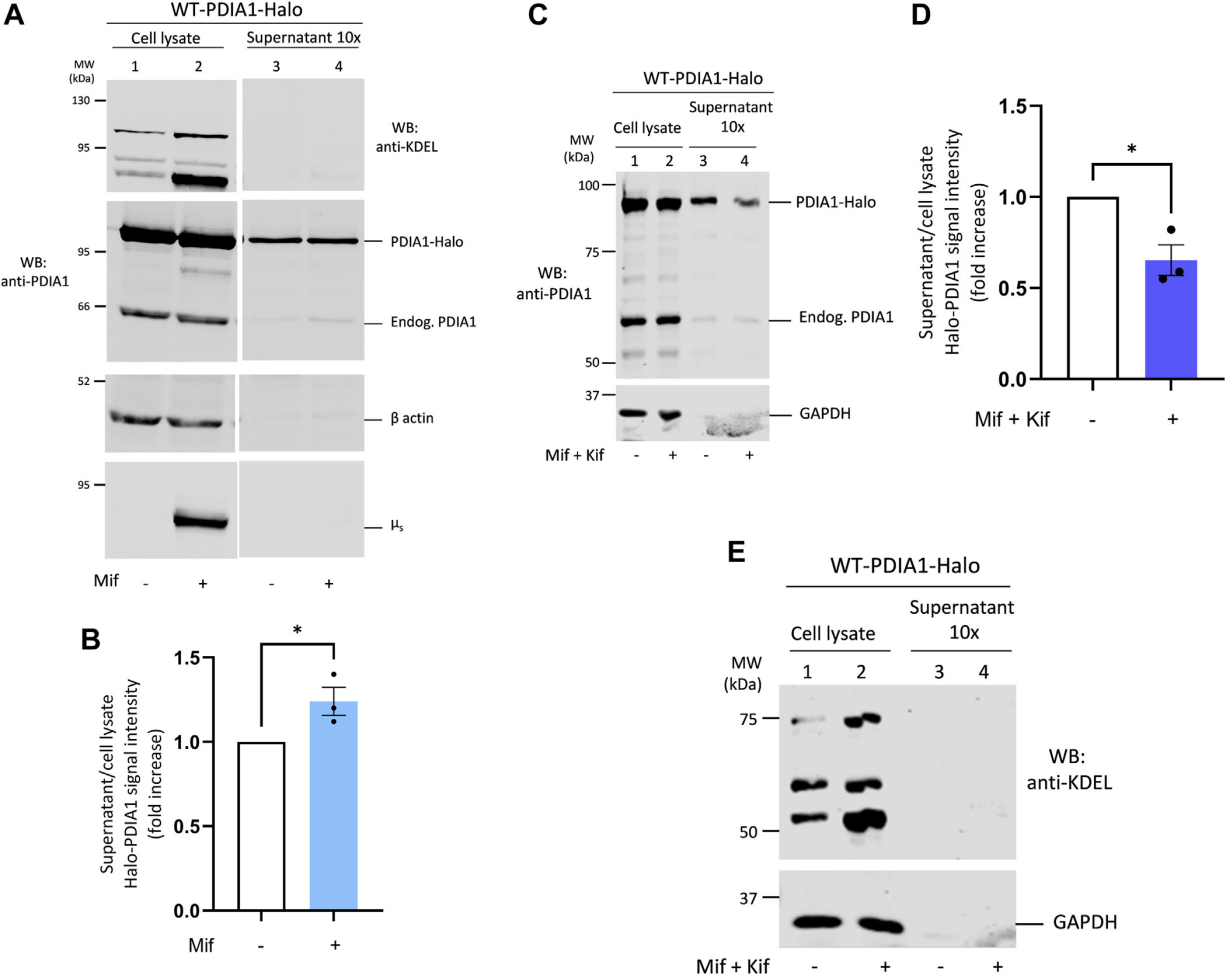
**Slightly different effects of adaptive or maladaptive UPR on PDIA1 secretion. HeLa cells expressing secretory Ig-μ**_**s**_**chains under control of mifepristone (Mif) were transiently transfected with WT-PDIA1**. *A* and *B*, forty-eight hours after transfection, cells were induced for 16 h with Mif alone to induce an adaptive UPR. In panels (*C*–*E*) samples were prepared in the same way. The simultaneous addition of Kifunensin (21.5 μM) blocks μ_s_ ER-associated degradation, causing maladaptive UPR (*C*–*E*). Noninduced cells served as controls. *A*, aliquots of cell lysates (corresponding to 0.1 × 10^6^ cells) and supernatants (corresponding to 1 × 10^6^ cells) were collected. After supernatants concentration through TCA precipitation, both were resolved by reducing SDS-PAGE (8% polyacrylamide gel) followed by immunoblotting with the indicated antibodies. All lanes come from a single gel. Whole blots are shown in Fig. S12. *B*, the graph shows a quantification of secreted PDIA1 relative to its intracellular pool. Bars represent means ± SEM. Statistical analysis was performed using unpaired *t* test. ∗*p* = 0.0449 (n = 3). *C* and *E*, samples were prepared as described in panel (*A*) and blot membranes decorated with indicated antibodies. *D*, graph as in panel (*B*). Bars represent means ± SEM. Statistical analysis was performed using unpaired *t* test. ∗ *p* = 0.0146 (n = 3). Kif, Kifunensin; PDIA1, protein disulfide isomerase-A1; UPR, unfolded protein response.

Recently, cancer-associated thrombosis was linked to an unfolded protein response (UPR)-dependent increased release of prothrombotic vesicles (38). Relocalization of selected ER chaperones to the cell surface could be part of cell autonomous as well as cell nonautonomous adaptive mechanism(s) in response to proteotoxic stress. Thus, to investigate whether PDIA1 release is exaggerated by an ongoing UPR, we exploited an established model in which physiological ER stress is triggered by the inducible bulk expression of orphan secretory Ig-μs chains (39, 40). The amount of secreted WT-PDIA1 after 16h of adaptive ER stress increased compared to unstressed cells (Fig. 7, *A* and *B*). β actin and Ig-μs chains themselves (a target of BiP-dependent retention) were barely detectable in the SNs, arguing against cell damage or death as the only source of secreted PDIA1. In this model, the ER-associated degradation (ERAD) pathway is functional, and there is an ER homeostatic readjustment to μ_s_ expression (39). To induce a failure of this readjustment, μ_s_-expressing cells were exposed to Kif to block ERAD of glycoproteins generating a model of unresolved ER stress (40). Under these stressful conditions, the amount of secreted WT-PDIA1 in Kif-treated cells decreased compared to unstressed cells, arguing in favor of specific release mechanisms rather than cell damage/death (Fig. 7, *C* and *D*). Accordingly, GAPDH and other KDEL-bearing proteins were undetectable in the SNs (Fig. 7*E*). Secreted Glyco-PDIA1 remained Endo-H sensitive (Fig. S7) indicating that its secretion was not due to perturbed KR retrieval. Thus, escape of ER chaperones is unlikely to reflect oversaturation of KR retrieval machinery. Accordingly, cell-surface expression of ER chaperones often precedes their intracellular increase upon ER stress (41). Active secretion should be invoked to explain an increase of extracellular PDIA1 in the first phases of an UPR. The homeostatic failure ensuing upon ERAD inhibition could dampen PDIA1 secretion *via* excessive client binding. Detailed analyses of pathophysiological models of PDIA1 release, like vascular EC, are needed to further dissect the underlying mechanisms.

## Concluding remarks

**Figure 8:**
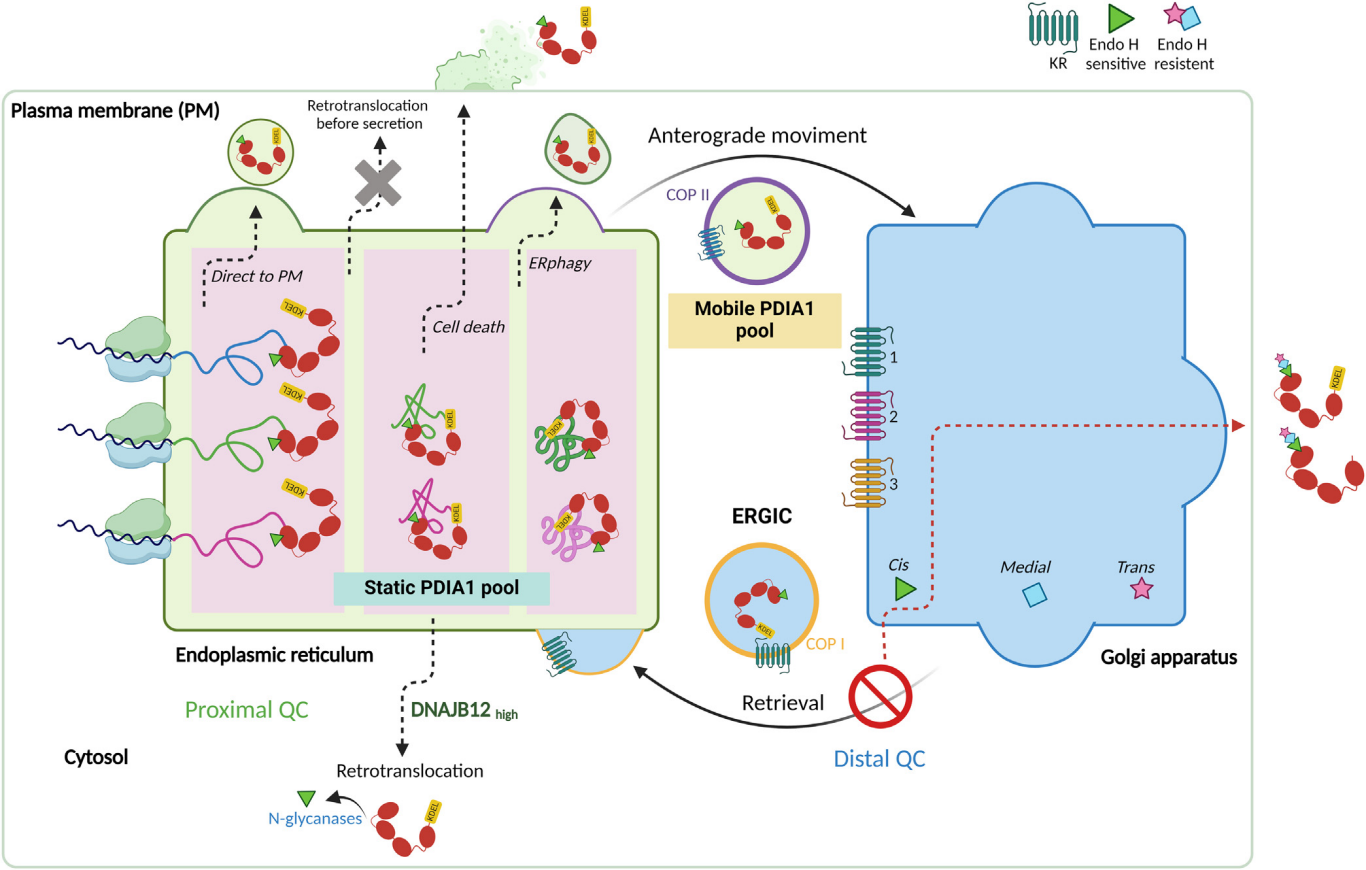
**Mechanisms of PDIA1 translocation.** The cartoon summarizes a model showing mechanisms of PDIA1 translocation that emerge from our studies. Original image created with BioRender. PDIA1, protein disulfide isomerase-A1.

Loss of membrane integrity remains the easiest way to explain the externalization of ER resident proteins. Yet, active secretion was unequivocally demonstrated for at least two of them, ERp44 and AGR2, by the different posttranslational modifications of extracellular molecules (19, 26). Our reporter yielded important mechanistic information on PDIA1 externalization (Fig. 8). The presence of Endo-H sensitive glycans on extracellular Glyco-PDIA1 molecules implies that they entered the ER and were not refluxed to the cytosol. As to how they reach the extracellular space, however, remains unclear: ER-plasma membrane contacts, ER-phagy, and delivery to secretory lysosomes could be involved, although the latter mechanisms did not account for PDIA1 secretion in HeLa cells since bafilomycin, which impairs lysosome acidification, did not affect significantly PDIA1 secretion (Fig. S8). In addition, secretory lysosomes were shown to play a minor if any role in endothelial cells (9). Finally, even the simplest interpretation, that is cell damage/death, would imply some selectivity, as PDIA1 and other proteins are more abundant than lactate dehydrogenase or GAPDH. It is tempting to speculate that organelle-specific damage associated molecular patterns evolved to coordinate non-cell-autonomous responses to stress and/or inflammation. Additional approaches are required to clarify how some ER residents change their address.

## Experimental procedures

### Reagents and antibodies

Unless otherwise specified, all reagents used were purchased from Sigma-Aldrich. Primary antibodies used in this study were as follows: PDIA1 (Invitrogen, MA3–019 1:1000), KDEL (Enzo Lifescience, SPA-827 1:1000), Halo-tag (Promega, G9281 1:1000), GAPDH (Abcam, ab8245 1:000), Ig-μ-Alexa Fluor 647 (Invitrogen Molecular Probes 1:1000), Myc-tag (Cell Signaling, 2278 1:1000), ERp44 (36C9, in-house, 1:500), β actin (Sigma, A2228 1:1000), DNAJB12 (Invitrogen, PA5-52720), DNAJB14 (Invitrogen, PA5-106326), and tubulin (Abcam, ab52866). Alexa-conjugated secondary antibodies (Invitrogen 1:1000). Fluorescent secondary antibodies (1:10000) were purchased from Odyssey LI-COR. Other reagents were purchased as follows: JetPEI (Euroclone), Lipofectamine RNAiMAX (Invitrogen), Opti-MEM (Gibco, Invitrogen), Endo-H (New England Biolabs), PNGase-F (New England Biolabs), TMR Direct Halo ligand (Promega, G2991), TriFast (Euroclone), Magne HaloTag Beads (Promega, G7281), and HaloTEV Protease (Promega, G6601).

### Cells, plasmids, small interfering RNAs, and transfection procedures

HeLa cells S3 were obtained from ATCC and were cultured in Dulbecco's modified Eagle's medium (Gibco, Invitrogen) supplemented with 10% fetal bovine serum (Euroclone), glutamine (2 mM), and penicillin/streptomycin.

The human PDIA1-Halo construct was synthesized in pCDNA 3.1 (+) vector. The Halo-tag was inserted between amino acids E497 and D498, upstream of the KDEL sequence. Site-directed mutagenesis was performed on PDIA1-Halo to introduce the mutation D109T using the following primers: FW- 5′-CAGAAACGGCACTACAGCCTCCCCTAAG-3′ and RV- 5′-CTTAGGGGAGGCTGTAGTGCCGTTTCTG-3′. To remove the KDEL C-terminal sequence, a stop codon was inserted between the Halo and the KDEL using the following primers: FW- 5′- CTGTCCACACTGGAGATCTGATGATGACCAGAAGGCC and RV-5′- GGCCTTCTGGTCATCATCAGATCTCCAGTGTGGACAG-3′. To generate PDI KDEL variants, site-directed mutagenesis was performed on PDIA1-Halo using the following primers: DDEL FW-5′- GACCAGAAGGCCGTGGACGATGAGCTGTG-3′ and RV-5′-CACAGCTCATCGTCCACGGCCTTCTGGTC-3′; ADEL FW-5′-GACCAGAAGGCCGTGGCGGATGAGCTGTG-3′ and RV-5′-CACAGCTCATCCGCCACGGCCTTCTGGTC-3′; HDEL FW-5′-GACCAGAAGGCCGTGCATGATGAGCTGTG-3′ and RV-5′- CACAGCTCATCATGCACGGCCTTCTGGTC. Mutations were validated by sequencing. Plasmids encoding Halo-Cμ234tp Ero1-α-Myc plasmid were described previously (31, 42). Plasmids encoding DNAJB12 (RC220679), DNAJB14 (RC204615), and pCMV6 vector (PS100001) were obtained from OriGene.

Cells were plated in 60 mm culture dishes to have about 50 to 60% confluence on the day of transfection. Following the manufacturer's instruction, cells were transfected with JET PEI reagent 48 h before analysis. Two to five microgram of Glyco-PDIA1 and 0.5 to 3 μg of WT-PDIA1 plasmid were used in all experiments, unless specified. Three microgram of Myc-Ero1α, 3 μg of DNAJB12, and 14 (alone) and 1.5 μg of each plasmid (when in combination) were used in all experiments. In silencing assays, cells were transiently transfected (3 μl duplexes, 20 μM or universal negative control) using Lipofectamine RNAiMAX following the manufacture’s instruction and analyzed after 72 h. When cells were both silenced and transfected, silencing was performed 24 h prior to transfection and cells assayed after 72 h from silencing. KDELR1 oligos: (1) GUUCAAAGCUACUUACGAU; (2) GGUGUUCACUGCCCGAUAU; (3) CCAACUACAUCUCACUCUA. KDELR2 oligos: (1) ACACAUCUAUGAAGGUUAUUU; (2) CAUGAUACCUUCCGAGUGGUU; (3) GAUCUGGCGCUUCUACUUUUU. KDELR3 oligo: AGUCGUGUCUGGAGUAGUA. All synthesized by Sigma.

### Secretion assays

Secretion assays were performed in minimal essential medium (Opti-MEM) using >10^6^ cells per time point. HeLa cells transiently transfected with corresponding plasmids were washed with PBS 1X then incubated with 2 ml of (Opti-MEM) for 4 h at 37% and 5% CO_2_. SNs were then collected and cleared at 1400 rpm for 5 min. The collected SNs were precipitated using trichloroacetic acid to concentrate proteins before loading in SDS-PAGE gels. Cells were detached using trypsin-EDTA and washed in PBS containing 10 mM NEM, and cell number was determined. Cell pellets were then lysed in RIPA buffer (150 mM NaCl, 50 mM Tris-HCl pH 7.5, 1% NP40, 0.5% Na Deoxycholate, 0.1% SDS) supplemented with complete protease inhibitors and 10 mM NEM. Postnuclear SNs were collected for further analysis. Lysates and SNs corresponding to >10^6^ cells were analyzed by Western blotting.

### Protein deglycosylation

Aliquots of postnuclear SNs (cell lysates, 10 μg of total protein) and trichloroacetic acid precipitated SN (corresponding to >10^6^ cells) were treated with Endo-H or PNGase-F following the manufacturer’s instructions and analyzed by Western blotting.

### Western blotting analysis

Protein samples were resolved in 6% or 8% SDS-PAGE gels, under reducing conditions, and transferred onto nitrocellulose membranes using wet transference. After transfer, the membrane was saturated with 5% milk in PBS 0.1% Tween and then sequentially incubated with primary and fluorescent secondary antibodies. Alexa Fluor (Invitrogen) conjugated with diverse fluorophores (647, 546 nm) or fluorescent-couple secondary antibodies from Odyssey LI-COR were used. The acquisition was performed at the correct wavelength using Typhoon FLA9000 Biomolecular Imager (GE Lifesciences) or detected by Odyssey System (LI-COR Biosciences). When required by the experimental design, densitometric analysis was performed with ImageJ software or LI-COR system program.

### Measurement of PDIA1 reductase activity

Reductase activity assays were performed using purified PDIA1-Halo from cell lysates of Glyco and WT-PDIA1-transfected HeLa cell. Halo-tagged protein purification was carried out following the manufacturer’s protocol (Promega). Briefly, cell lysates (2000 μg/μl) were added to equilibrated Magne HaloTag Beads (50 μl) and incubated for 90 min at room temperature with constant mixing. Using a magnetic stand, beads were washed twice, and then protein purification was performed through proteolytic cleavage of the HaloTag. Reductase assay buffer (0.1 M potassium phosphate pH: 7.4, containing 2 mM EDTA) was added to the washed beads combined with HaloTEV protease (5 μl) and incubated at room temperature for 90 min with constant mixing. After that, SNs containing cleaved PDIA1 were carefully removed. Reductase activity was carried out by incubation of the SNs (150 μl), 5 μM DTT, and 150 nM of di-eosin-GSSG. The increase in fluorescence was determined for 50 min by excitation at 520 nm and emission at 545 nm in a SpectraMax-M5. Reduction of 150 nM di-eosin-GSSG by 5 μM DTT served as a negative control. Three independent experiments were performed, and each sample was detected in duplicate. The probe di-eosin-GSSG was prepared as previously described (27, 28). Mock-transfected cells showed a residual reductase activity that was subtracted from Glyco and WT-PDIA1 activity values.

## Data availability

All data are contained within the main manuscript and supporting information.

## Supporting information

This article contains supporting information (39, 40).

## Conflicts of interest

The authors declare no conflict of interest with contents of this article.
