# Supplementary material for: Transport of protein disulfide isomerase from the endoplasmic reticulum to the extracellular space without passage through the Golgi complex
Source: J Biol Chem. 2024 Jul 4;300(8):107536. doi: 10.1016/j.jbc.2024.107536 (PMC11342103; doi:10.1016/j.jbc.2024.107536)
Supplement: Supporting information [file mmc1.docx]

**Supporting information**

**Transport of Protein Disulfide Isomerase from the Endoplasmic Reticulum to the Extracellular Space without Passage through the Golgi Complex**

Percillia Victoria Santos Oliveira^1,2^, Marco Dalla Torre^1^, Victor Debbas^2^, Andrea Orsi^1^, Francisco Rafael Martins Laurindo*^2^ and Roberto Sitia*^1^

^1^Division of Genetics and Cell Biology Vita-Salute San Raffaele University and IRCCS San Raffaele Scientific Institute, Milan, Italy

^2^Laboratorio de Biologia Vascular, LIM-64 (Biologia Cardiovascular Translacional), Instituto do Coração (InCor), Hospital das Clinicas HCFMUSP, Faculdade de Medicina, Universidade de São Paulo, São Paulo, SP, Brasil

**Running title**

Mechanisms of PDIA1 secretion

*Corresponding authors:

**Francisco Rafael Martins Laurindo, MD, PhD**

Email: francisco.laurindo@hc.fm.usp.br

**Roberto Sitia, MD, PhD**

Email: sitia.roberto@hsr.it

**Supplementary Materials and Methods**

*Non-radioactive Halo-ligand pulse chase*

HeLa cells transfected with Glyco-PDIA1ΔKDEL-Halo, WT-PDIA1ΔKDEL-Halo and ERp44ΔRDEL-Halo (6 µg each plasmid). After 48h equal amounts of cells (2x10^6^) were incubated for 1 h at 37 °C in complete medium supplemented with 30 µM 6-Chlorohexanol (a colorless Halo ligand), washed 3 times in PBS and incubated for 15 min at 37 °C with 100 nM TMR Direct Halo ligand (pulse). After washing in ice cold PBS containing 30 µM 6-Chloroexanol and 2 μg/mL puromycin, cells were incubated for the desired chase times at 37°C in Opti-MEM supplemented with 30 µM 6-Chlorohexanol and puromycin. 2x10^6^ cells were used per time point for secretion assays. Supernatants and lysates corresponding to 2x10^5^ cells were loaded on 8% gel and Fluorescent TMR Direct Halo ligand signal was detected directly on gel by Typhoon FLA 9000 scanner (GE) and analyzed using ImageJ software.

*Radioactive pulse chases*

HeLa cells transfected with Glyco-PDIA1 plasmid (6 µg) were resuspended at 10^7^ cell/mL in DMEM without methionine and cysteine supplemented with 1% dialyzed FBS. After 20 min at 37°C, cells were pulsed for 15 min with 33 μCi/10^6^ cells of ^35^S-labeled methionine and cysteine (EasyTag, Perkin Elmer), washed twice in PBS and chased in Opti-MEM with 2.5 mM unlabeled aminoacids for the desired chase times. For analysis of total radioactivity cell lysates were precipitated (IP) with anti-PDIA1 (RL90) linked to G protein beads. IP samples were subjected to different washing steps to get rid of associated proteins: firstly, with solution ‘A’ (500 mM NaCl, 10 mM Tris-HCl pH 7.5, 0.5% NP40, 0.05% SDS), then in STN (150 mM NaCl, 10 mM Tris-HCl pH7, 0.025% NP-40) supplemented with 100 mM DTT and once more in STN buffer supplemented with 100 mM NEM. Immunoprecipitated material was eluted from the beads in reducing Laemmli buffer, and resolved by 8% poly-acrylamide gels. Proteins were transferred onto nitrocellulose membrane and then dried on 3MM paper. Membranes were exposed to LE storage phosphor screen (Amersham). Radioactive signal acquired on a Typhoon FLA 9000 scanner (GE).

*Immunofluorescence analysis*

HeLa transfectants expressing WT-PDIA1 and Glyco-PDIA1 were incubated for 4 h with TMR-Direct Halo ligand (100 nM) then fixed in 4% PFA, permeabilized in PBS/0.1% TritonX-100, blocked with PBS/ 10% FCS for 1 h at room temperature and labelled with rabbit anti-CRT (Sigma, C4606, 1:500) and mouse anti-GM130 (Becton-Dickinson, 710823, 1:500) antibodies. Fluorescent images were obtained using a GE Healthcare DeltaVision™ Ultra microscope and 60X objective lens.

*Quantitative RT-PCR*

Total RNA was extracted from cells with 1 mL of phenol-based reagent TriFast for RNA extraction according to the manufacturer’s instructions. Retro-transcription to cDNA was performed with M-MLV Reverse Transcriptase and random primers (Promega) following the manufacturer’s protocol for 2 μg RNA. Real-time semi-quantitative PCR was performed with iTaq Universal SYBR Green Supermix (Bio-Rad) using the following primers: Hs_KDELR1_1_SG QuantiTect Primer Assay (QT00090811) Hs_KDELR2_1_SG QuantiTect Primer Assay (QT00092715) Hs_KDELR3_1_SG QuantiTect Primer Assay (QT00097790). All from Qiagen. GAPDH forward primer (TGAAGGTCGGAGTCAACGGATTT), GAPDH reverse primer (CATGTAAACCATGTAGTTGAGGT). Each reaction was performed in duplicate in a total volume of 10 μL. Amplification was performed with the BioRad CFX96TM System. GAPDH was chosen as a reference gene for relative quantifications. For all experiment conditions, the ∆Ct was calculated by subtracting the Ct of the reference gene from the Ct of the gene of interest. The values obtained were then used to calculate the ∆∆Ct by subtracting the ∆Ct of the control condition from the other conditions. These numbers were finally used to calculate the fold gene expression value (2-∆∆Ct) often used in literature.

**Supplementary Figures**

**
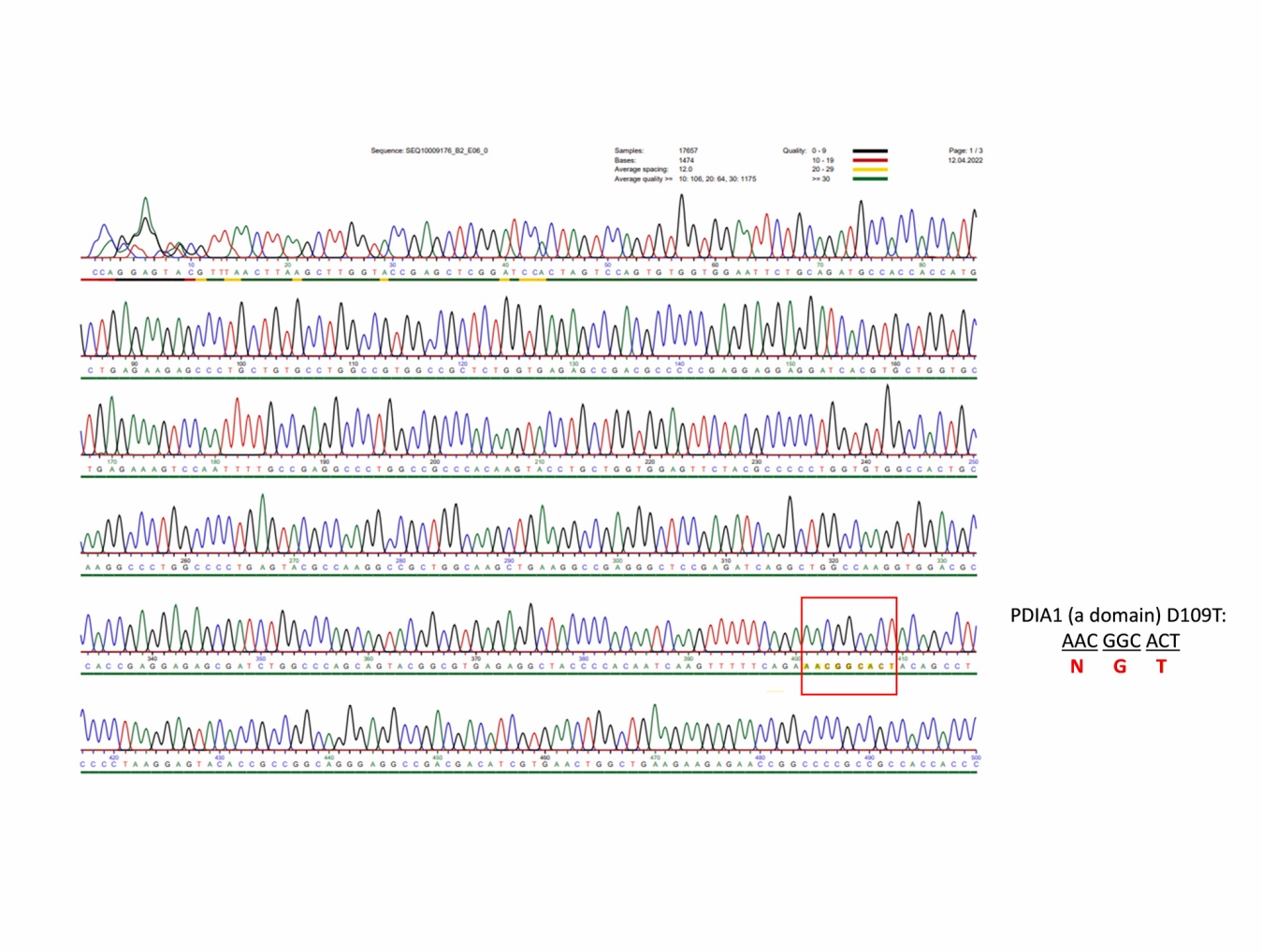
**

**Figure S1. Sequencing data confirming Glyco-PDIA1 mutagenesis.** Using site directed mutagenesis the glutamate-109 (D109) residue was replaced to a threonine-109 (T109) through a codon changing (GAT to ACT).


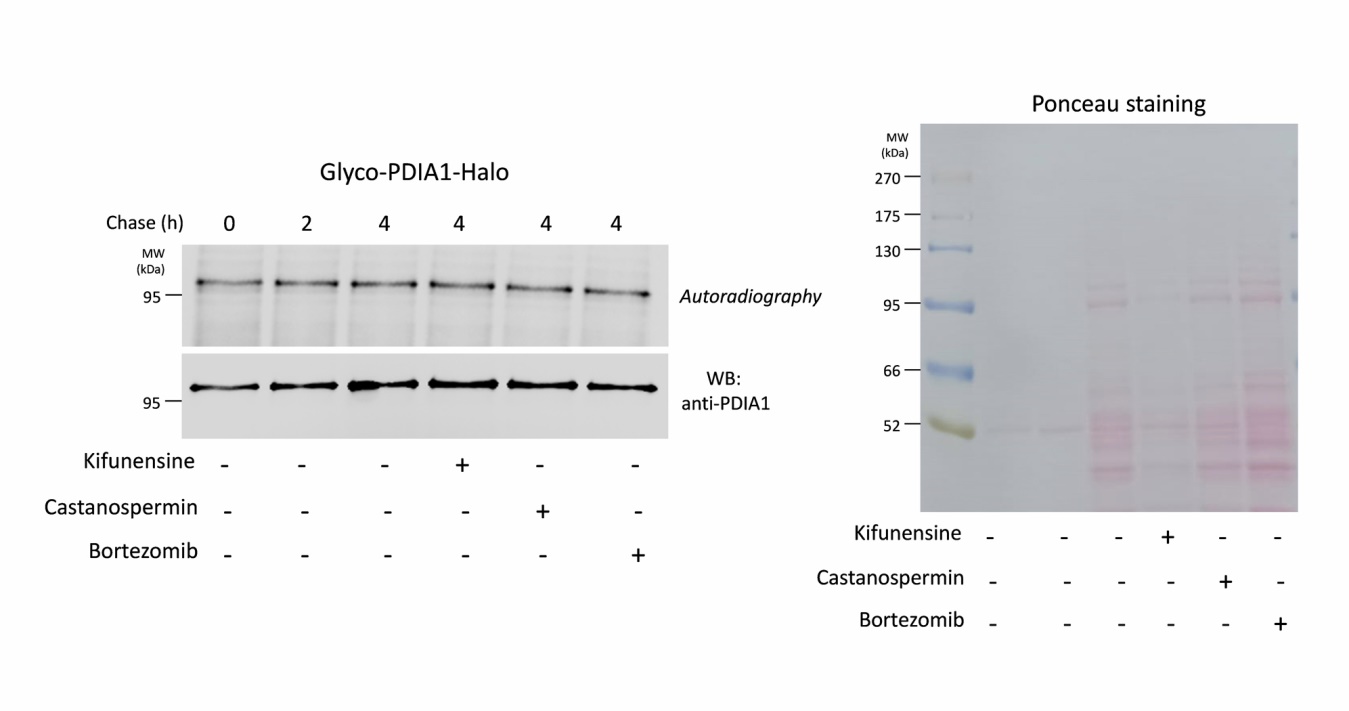


**Figure S2. The presence of a N-glycan does not impair the stability of Glyco-PDIA1.** HeLa cells transiently expressing Glyco-PDIA1 were pulsed-labeled with ^35^S-labeled methionine and cysteine for 15 min and chased for the indicated times before cell lysis. Glyco-PDIA1 was immunoprecipitated with anti-PDIA1. Immunoprecipitates were resolved by SDS-PAGE, transferred to nitrocellulose filters and subjected to autoradiography to detect newly made proteins. Then, membranes were immune decorated with anti-PDIA1 to detect the whole pool of PDIA1 molecules, including the non-radioactive pre-existing molecules or those synthesized during the chase (n=2). Ponceau staining is used as loading control.


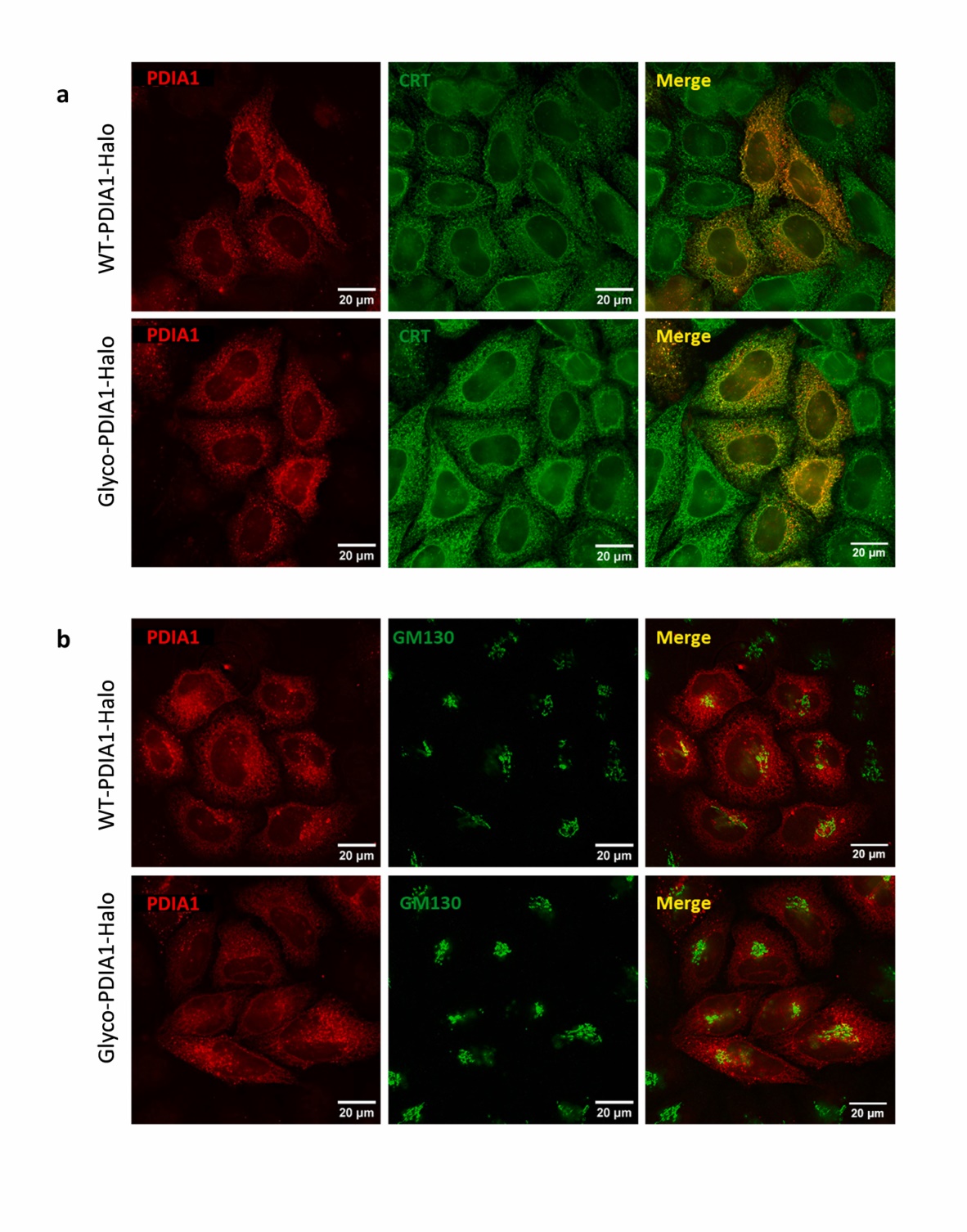


**Figure S3. WT and Glyco-PDIA1 accumulate mainly in the ER**. HeLa cells transiently expressing Glyco-PDIA1 and WT-PDIA1 were incubated for 4h with TMR (tetramethylrhodamine) Halo ligand (Red) and then fixed and labeled with anti-calreticulin (CRT) (green) (**a**) and anti-GM130 antibodies (green) (**b**) (n=3). Note the overlap of the anti-CRT and Halo signals. Scale bar 20 µm.


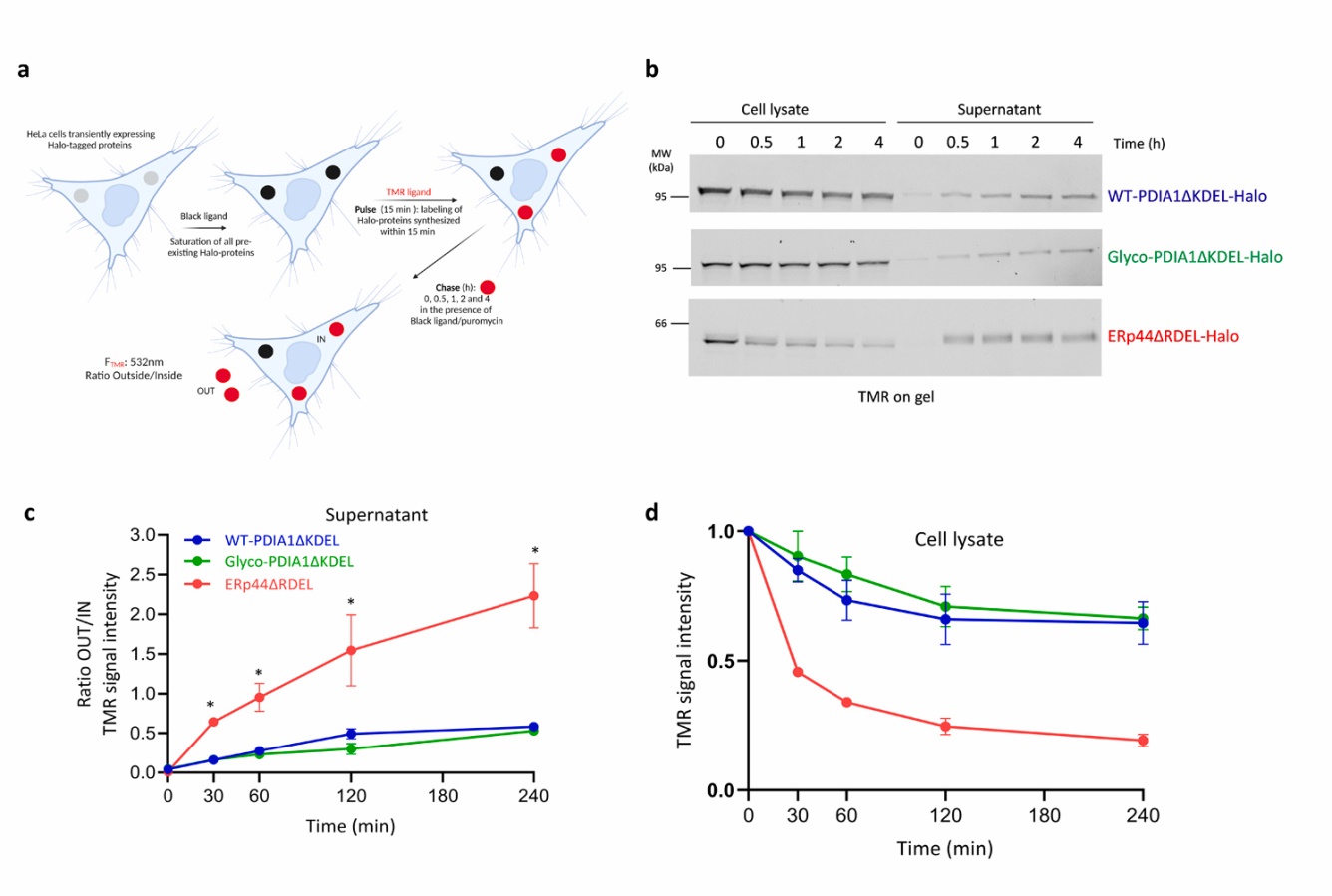


**Figure S4. Non-radioactive Halo pulse-chase secretion assays.** **a.** Scheme of the experiment. HeLa were transiently transfected with WT-PDIA1ΔKDEL, Glyco-PDIA1ΔKDEL and ERp44ΔRDEL. Forty-eight hours after transfection, all pre-existing molecules were saturated with a colorless Halo ligand. After thorough washing, cells were incubated for 15 min with a fluorescent ligand (tetramethylrhodamine, TMR, red) to label proteins synthesized during that period, washed again and chased for the indicated times in the presence of colorless Halo ligand and puromycin. At different time points, cell lysates and supernatants were resolved electrophoretically and the Halo/TMR fluorescent signals detected in gels (532 nm). Original image created with BioRender. **b.** Aliquots of cell lysates and supernatants corresponding to 1x10^6^ cells were resolved electrophoretically under reducing conditions, and gels were directly analyzed by fluorography (Thypoon, 532 nm). **c-d.** The graphs show the densitometric quantification of 3 independent experiments like the one showed in (**b**). **c.** The supernatant/intracellular ratio of the TMR-labeled signals was calculated for each time point. Statistical analysis was performed using non-parametric Mann-Whitney test; *p=0.05. The asterisks are referred to the comparison of WT-PDIA1ΔKDEL with ERp44ΔRDEL. **d.** Densitometry quantification of protein disappearance from the lysates (level of TMR signal at each time chase with respect to time 0). Note that at the end of the chase (4 h) almost all ERp44 was secreted, whereas a considerable amount of PDIA1 was still present intracellularly (n=3).


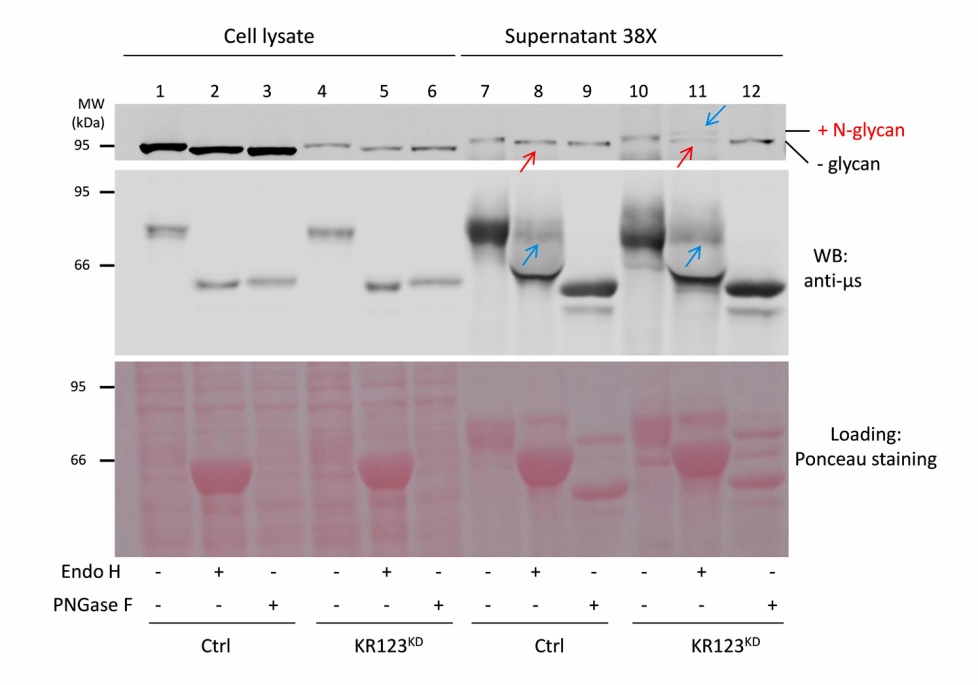


**Figure S5. KR silencing does not affect secreted IgM N-glycosylation.** KDEL receptors 1, 2 and 3 were silenced in HeLa cells inducible to secrete IgM (1, 2) and forced to transiently express Glyco-PDIA1. Forty-eight hours after transfection and 72 h after silencing, cells were incubated with or without Mifepristone (Mif) 0.5 nM for 16h to induce polymeric assemble of λ and µ_s_ IgM subunits. After that, cells were washed and incubated with Opti-MEM for 4 h. Aliquots of cell lysates (corresponding to ~20x10^3^ cells, 10 µg) and supernatants (corresponding to 1x10^6^ cells) were collected. After supernatants concentration through TCA precipitation both were treated with Endo-H and PNGase F. Samples were resolved by reducing SDS-PAGE (8% polyacrylamide gel) and blots were decorated with anti- IgM (µ chain) and anti-PDIA1 (n=2). The red arrow points to Endo-H sensitive PDIA1 molecules and the blue arrow points to Endo-H resistant PDIA1 molecules.


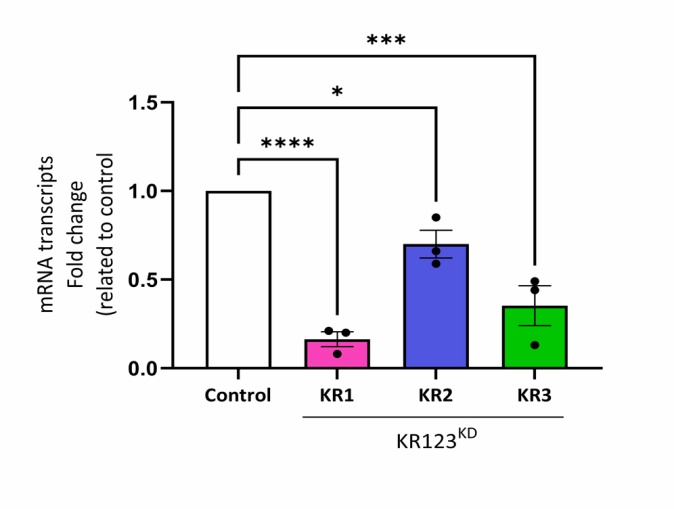


**Figure S6. KDEL receptor (KR) transcript levels upon silencing.** HeLa cells were silenced with KDEL receptors 1, 2 and 3-specific triplex or Control (Ctrl) and 72 h after silencing cells were collected and KRs mRNA levels accessed by RT-PCR. All data were normalized to GAPDH levels. Fold change is related to siRNA control (CTRL). Bars represent means ± SEM. *p=0.0440, ***p<0.0006, ****p<0.0001, (n=3).


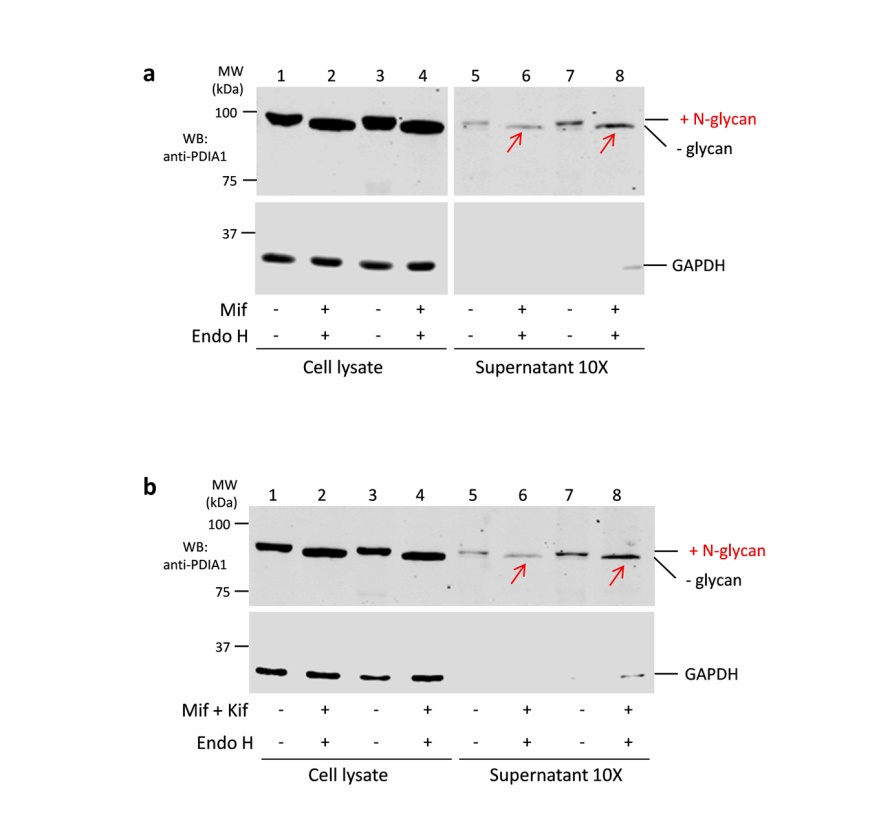


**Figure S7. Glyco-PDIA1 remains Endo-H sensitive upon ER stress.** HeLa cells expressing secretory heavy chain of IgM (µ_s_) under control of Mifepristone (Mif) were transiently transfected with Glyco-PDIA1. Forty-eight hours after transfection, cells were induced with Mif (**a**) or a combination of Mif plus Kifunensine (Kif) as described in Fig. 7 (**b**) for 16 h. Non-treated HeLa cells served as controls. After that, cells were washed and incubated with Opti-MEM for 4 h. Aliquots of cell lysates (corresponding to ~2x10^4^ cells) and supernatants (~1x10^6^ cells) were treated with Endo-H. resolved by reducing SDS-PAGE and blotted with anti-PDIA1 and anti-GAPDH (n=2). The red arrow points to Endo-H sensitive PDIA1 molecules.


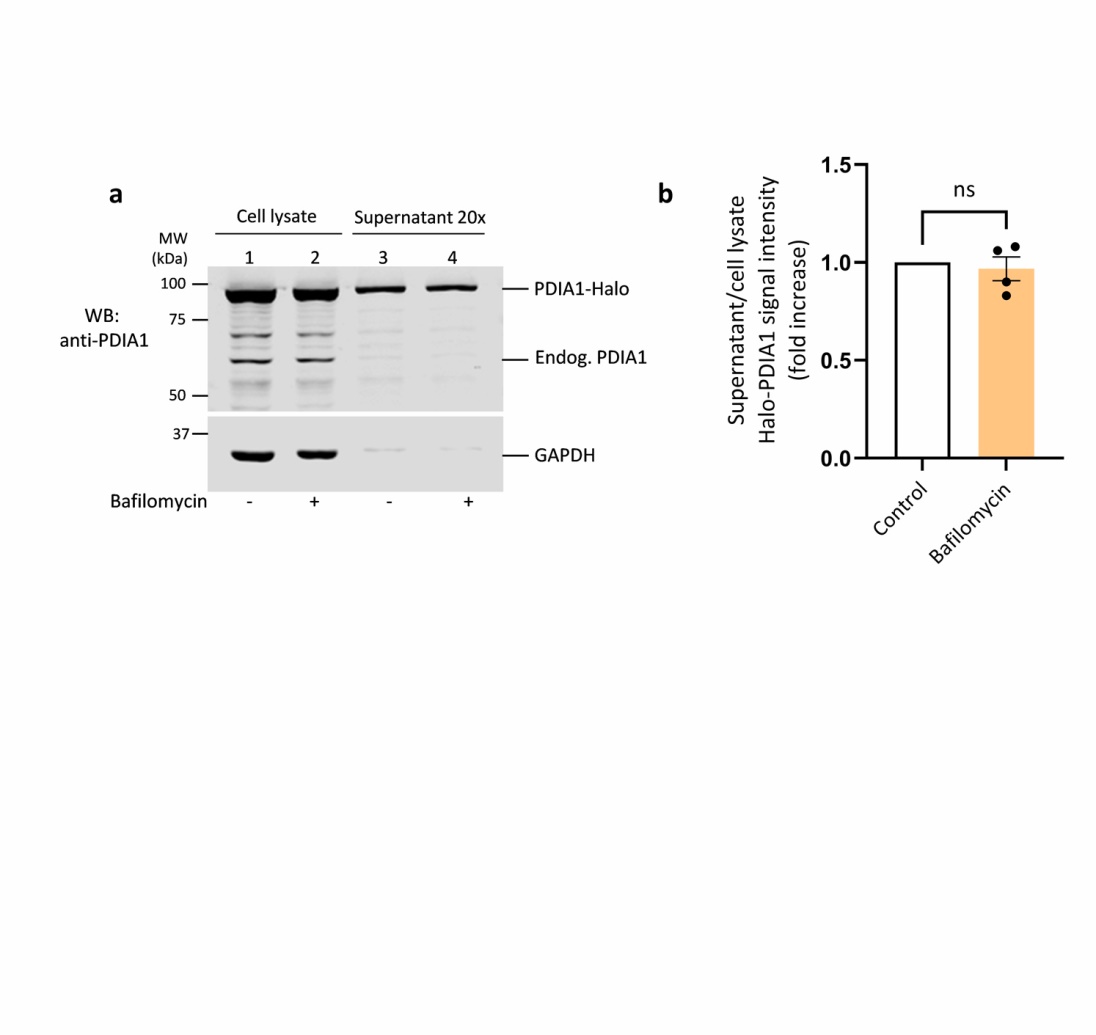


**Figure S8. Evidence that secretory lysosome does not contribute to PDIA1 secretion.** HeLa cells expressing WT-PDIA1-Halo were washed and incubated in Opti-MEM for 4 h. **a.** Aliquots of cell lysates (corresponding to 0.1x10^6^ cells) and supernatants (corresponding to 1.5x10^6^ cells) were collected. After supernatant concentration through TCA precipitation, both were resolved and immunoblotted with anti-PDIA1 or anti-GAPDH. **b.** The graph shows a quantification of secreted PDIA1 relative to its intracellular pool. Bars represent means ± SEM. Statistical analysis was performed using unpaired t test, ns: non-significant (n=4).


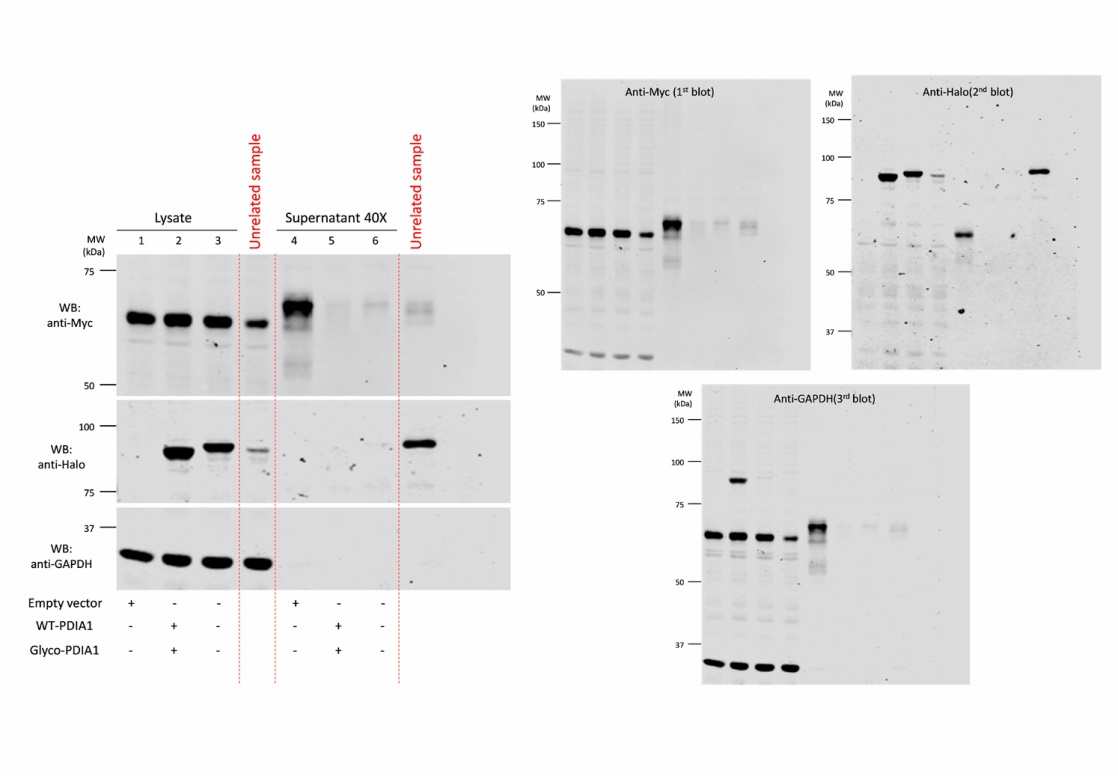


**Figure S9. Original membrane blots from Figure 2g.**

**
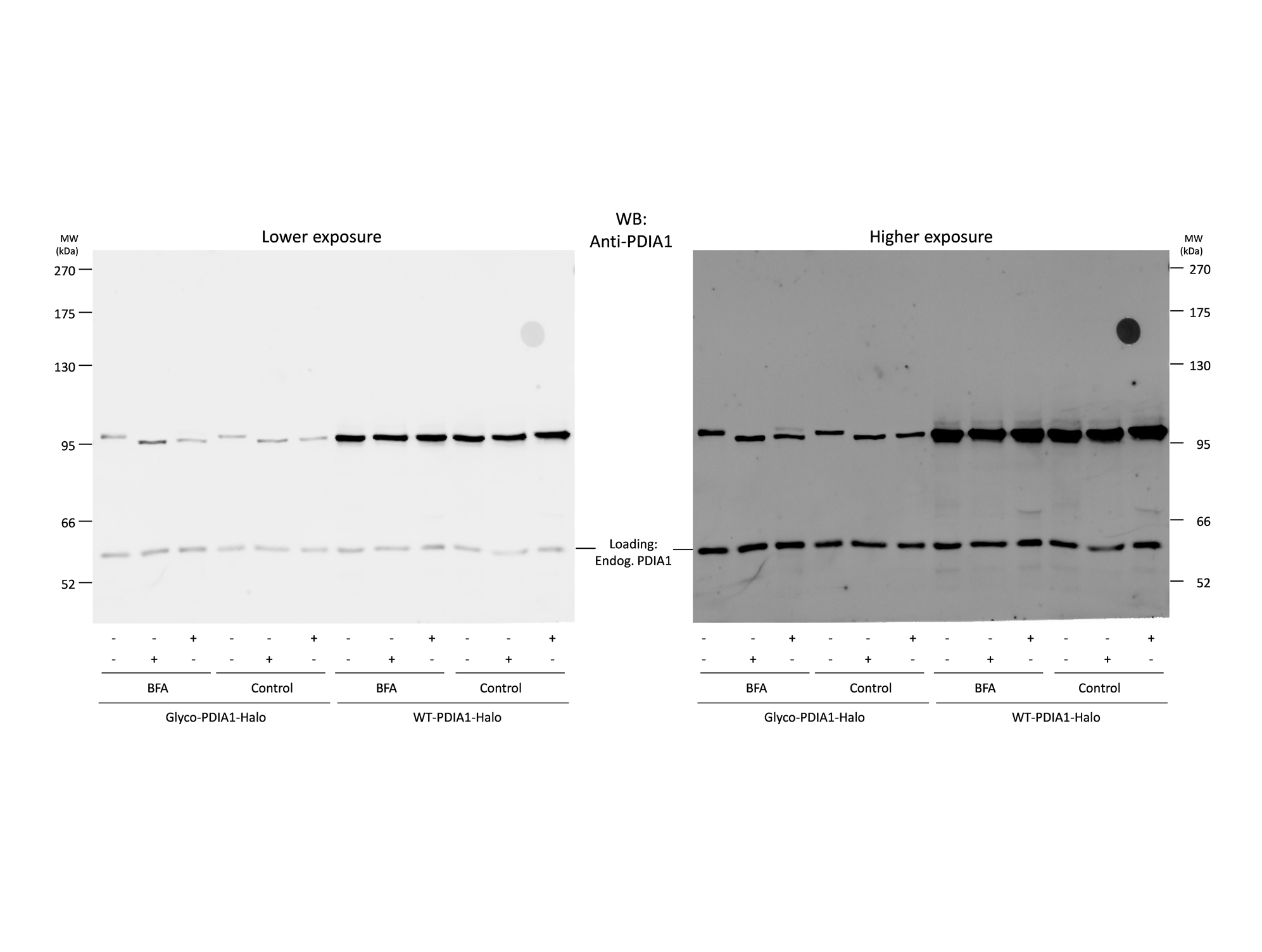
**

**Figure S10. Original membrane blots from Figure 3a.**

**
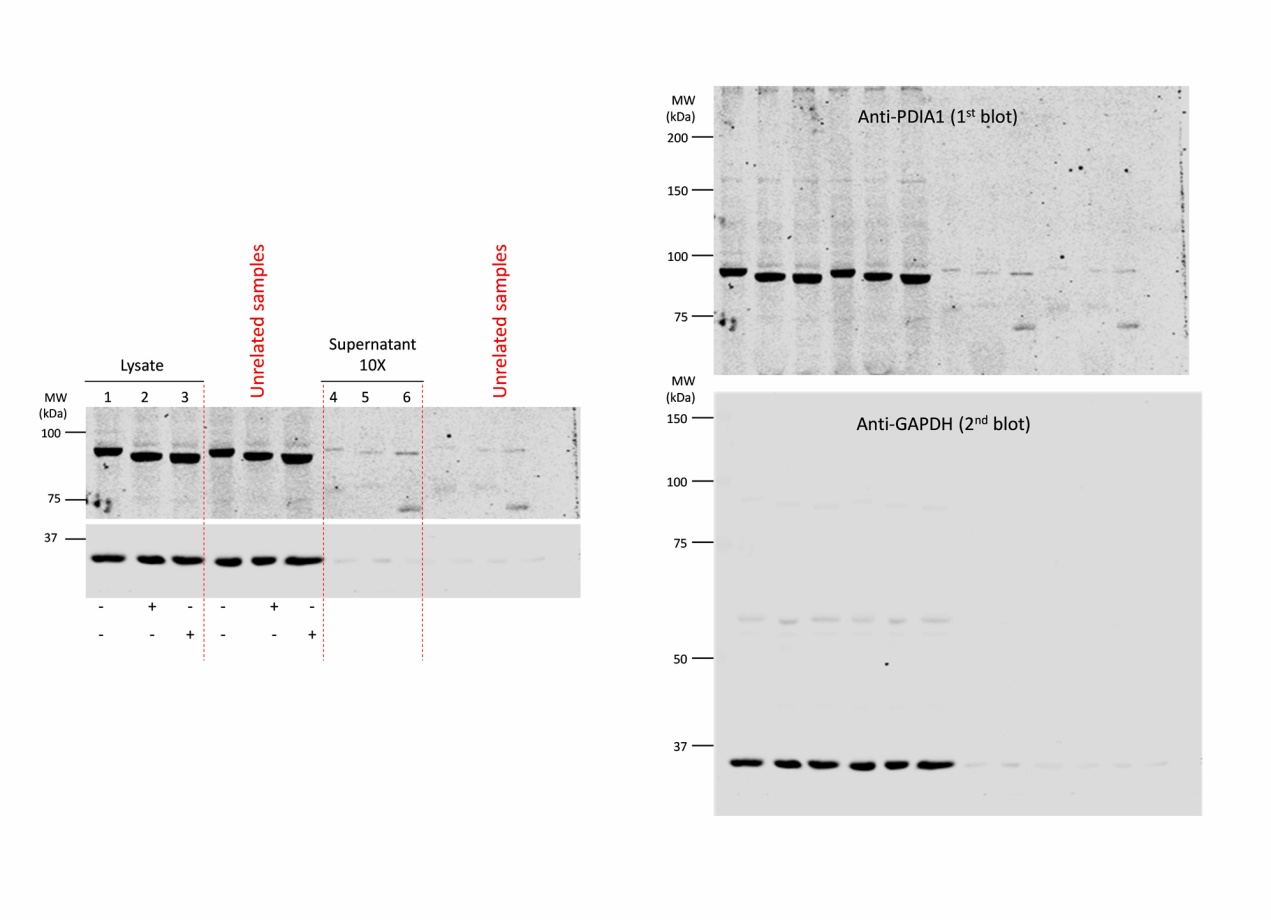
**

**Figure S11. Original membrane blots from Figure 6d.**

**
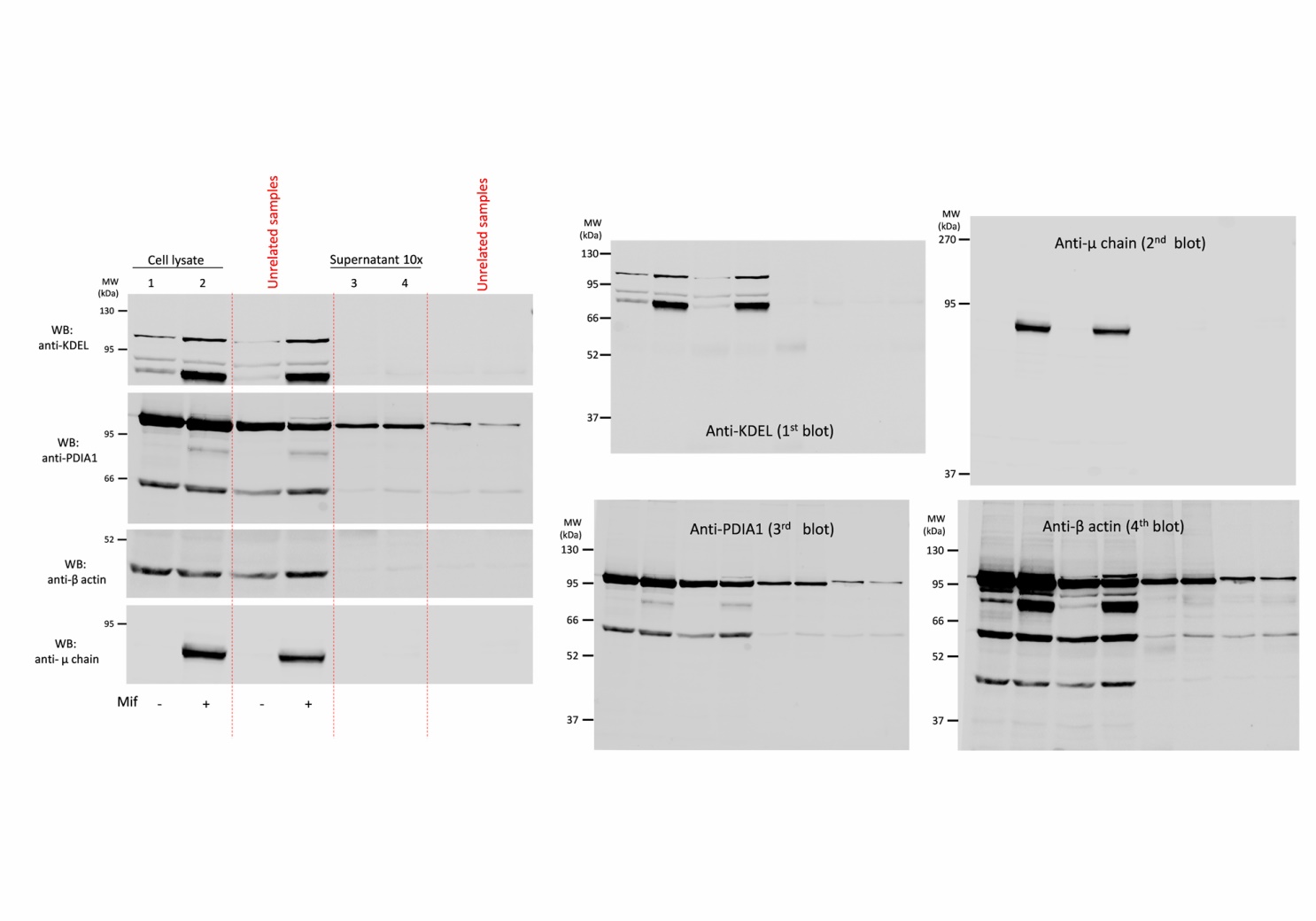
**

**Figure S12. Original membrane blots from Figure 7a.**

**
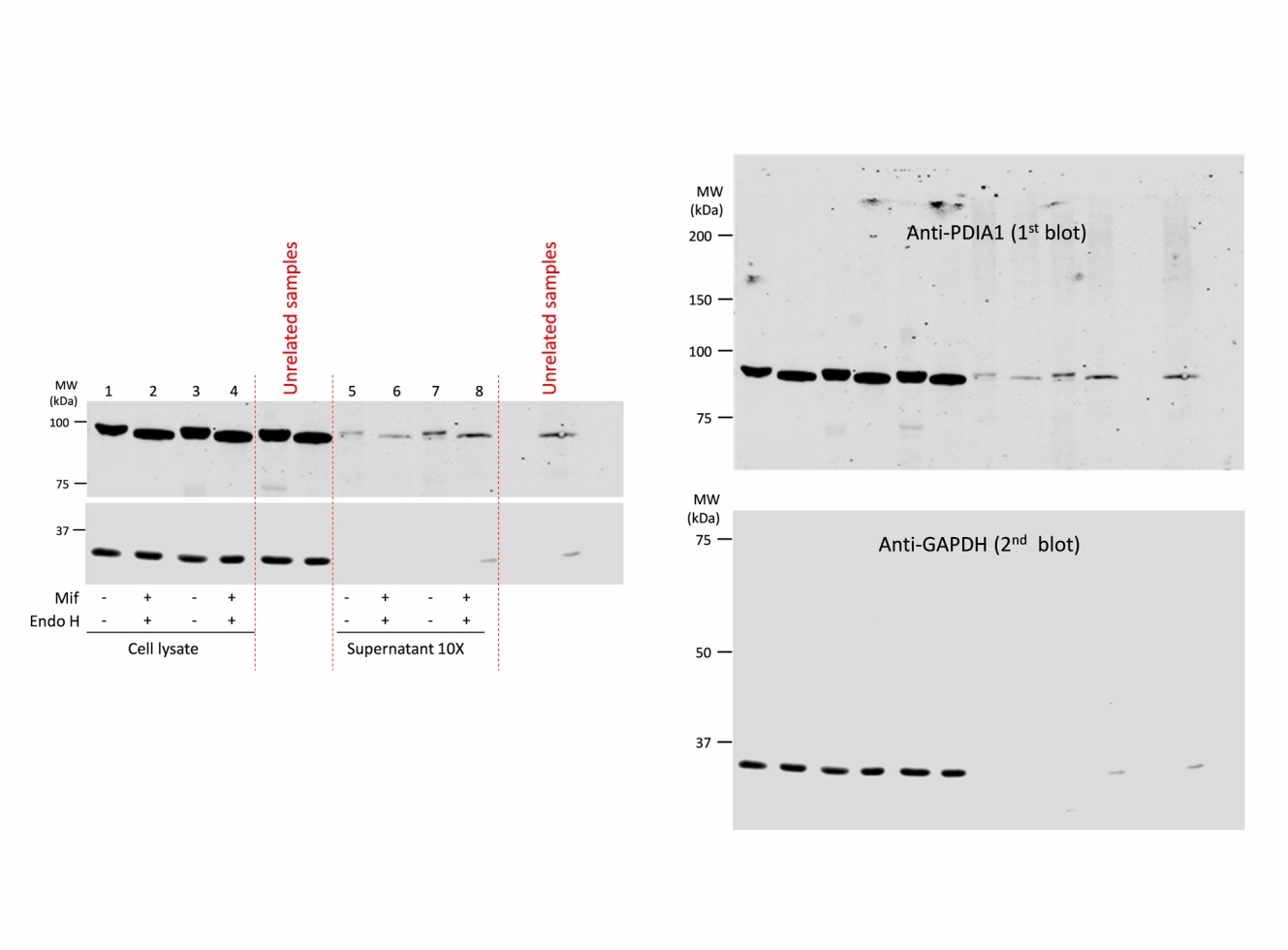
**

**Figure S13. Original membrane blots from Figure S7a.**

**References**

1. Bakunts, A., Orsi, A., Vitale, M., Cattaneo, A., Lari, F., Tade, L., Sitia, R., Raimondi, A., Bachi, A., and van Anken, E. (2017) Ratiometric sensing of BiP-client versus BiP levels by the unfolded protein response determines its signaling amplitude. *Elife* **6**

2. Vitale, M., Bakunts, A., Orsi, A., Lari, F., Tade, L., Danieli, A., Rato, C., Valetti, C., Sitia, R., Raimondi, A., Christianson, J. C., and van Anken, E. (2019) Inadequate BiP availability defines endoplasmic reticulum stress. *Elife* **8**
